# The role of bracket fungi in creating alpha diversity of invertebrates in the Białowieża National Park, Poland

**DOI:** 10.1002/ece3.7495

**Published:** 2021-03-31

**Authors:** Anna K. Gdula, Szymon Konwerski, Izabella Olejniczak, Tomasz Rutkowski, Piotr Skubała, Bogna Zawieja, Dariusz J. Gwiazdowicz

**Affiliations:** ^1^ Faculty of Forestry and Wood Technology Poznań University of Life Sciences Poznań Poland; ^2^ Faculty of Biology Natural History Collections Adam Mickiewicz University Poznań Poland; ^3^ Institute of Biological Sciences Cardinal Stefan Wyszyński University Warsaw Poland; ^4^ Faculty of Natural Sciences University of Silesia in Katowice Katowice Poland; ^5^ Department of Mathematical and Statistical Methods Poznań University of Life Sciences Poznań Poland

**Keywords:** Arachnida, biodiversity, Collembola, Insecta, richness, sporocarps

## Abstract

Bracket fungi are seen mainly as the cause of economic losses in forestry, and their role as creators of biodiversity is relatively poorly understood. The aim of the study was defining the manner in which the degree of decay (DD) of the fruiting bodies determines the character of the invertebrate assemblages colonising them. The effect of this group of fungi on the modification of biodiversity of invertebrates (Aranae, Opiliones, Pseudoscorpionida, two groups of mites—Mesostigmata and Oribatida, and Collembola and Insecta) was investigated by analyzing 100 fruiting bodies of 10 species of bracket fungi divided into four DD classes. The material was collected at Białowieża National Park, which is considered to be the largest area of natural forests in the North European Plain. 16 068 invertebrate individuals classified into 224 species were obtained. Oribatid mites (12 543 individuals) constituted the largest group of individuals, which were classified into 115 species with the most numerous *Carabodes femoralis* (8,811 individuals). Representatives of this group of mites have been reported previously in the publications on bracket fungi; however, the contributions of Oribatida and other groups of invertebrates were not broadly compared. Moreover, the species such as *Hoploseius mariae* and *H. oblongus*, which were predominantly found in fruiting bodies of bracket fungi, have also been discerned. The invertebrate fauna differs depending on DD of the samples: In the more decayed samples, a higher number of both individuals and species were recorded compared to the samples with lower DDs; however, this trend proved to be nonlinear. The DCA and cluster analysis revealed a similarity of the invertebrate assemblages from the 2 DD and 4 DD samples. They also indicated that the group 3 DD differed the most from all the other samples. The indicator species analysis identified species characteristic to individual DDs: For group 1 DD, it was, for example, *Hoploseius oblongus;* for 2 DD—*Orchesella bifasciata*; and for 3 DD—*Chernes cimicoides*, while for 4 DD—*Dinychus perforatus*.

## Graphical abstract

From the viewpoint of forest management, wood‐decaying fungi are mainly perceived in terms of financial losses resulting from the deteriorating timber value. Nevertheless, these fungi create a unique microhabitat of decaying wood; hence, their fruiting bodies play a significant role in promoting biodiversity of many groups of invertebrates.

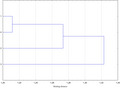

## 1 INTRODUCTION

From the viewpoint of forest management, wood‐decaying fungi are mainly noted in terms of financial losses resulting from the deteriorating timber value. Nevertheless, these fungi create a unique microhabitat of decaying wood; hence, their fruiting bodies play a significant role in promoting biodiversity of many groups of organisms, for example, woodpeckers (Butler et al., 2004; Conner et al., 1976; Jackson & Jackson, 2004), bats (Parsons et al., 2003), and small mammals (Bowman et al., 2000; Maser et al., 1978; Suter & Schielly, 1998). Invertebrates are an important group of organisms found in decaying wood and fruiting bodies of wood‐decaying fungi—this group is particularly interesting since many species choose to only colonize such insufficiently studied microhabitats (Gwiazdowicz, 2002; Makarova, 2002; O'Connell & Bolger, 1997a; Pielou & Verma, 1968). To date, studies have been undertaken to investigate various groups of invertebrates found in fruiting bodies of bracket fungi such as insects (e.g., Jonsell & Nordlander, 2004; Komonen, 2001), spiders (e.g., Ackerman & Shenefelt, 1973; Pielou & Pielou, 1968), or mites (e.g., Gwiazdowicz & Łakomy, 2002; Hågvar & Steen, 2013). Unfortunately, the knowledge of the subject is still insufficient and there is a lack of comprehensive studies covering all invertebrate groups and the character of their assemblages. Furthermore, there are almost no publications identifying factors, which determine the species composition and dependencies within those groups. In contrast to the studies investigating the fauna colonizing decaying wood, the publications concerning invertebrate assemblages which colonize fruiting bodies of bracket fungi neglect to analyze the degree of decay in bracket fungi. Studies have particularly been focusing on the fauna of invertebrates found in various fungal species (e.g., Gwiazdowicz & Łakomy, 2002; Makarova, 2002).

This study aimed to investigate the invertebrate fauna colonizing such a unique microhabitat as fruiting bodies of bracket fungi, as well as to identify how degree of decay (DD) of fruiting bodies determines the character of these assemblages. The research derives from previous studies conducted by Gwiazdowicz and Łakomy (2002) stating that the fungal species is not a key factor in determining the character of the invertebrate assemblage. Other research also showed that it was not the species of the fungus, but other factors (whether the fruiting bodies were alive or dead) that influenced the invertebrate communities inhabiting it (Thunes & Willasen, 1997). Also in the O’Connell and Bolger (1997a) research in which the invertebrate fauna inhabiting the fruiting bodies of 40 species of fungi was examined, characteristic faunas were not detected for any species or higher taxon of fungus. For this reason, a hypothesis was formulated that the invertebrate assemblages (both the number and the species’ richness) colonizing fruiting bodies of bracket fungi vary depending on their degree of decay (DD).

## 2 MATERIALS AND METHODS

### 2.1 Study area

**FIGURE 1:**
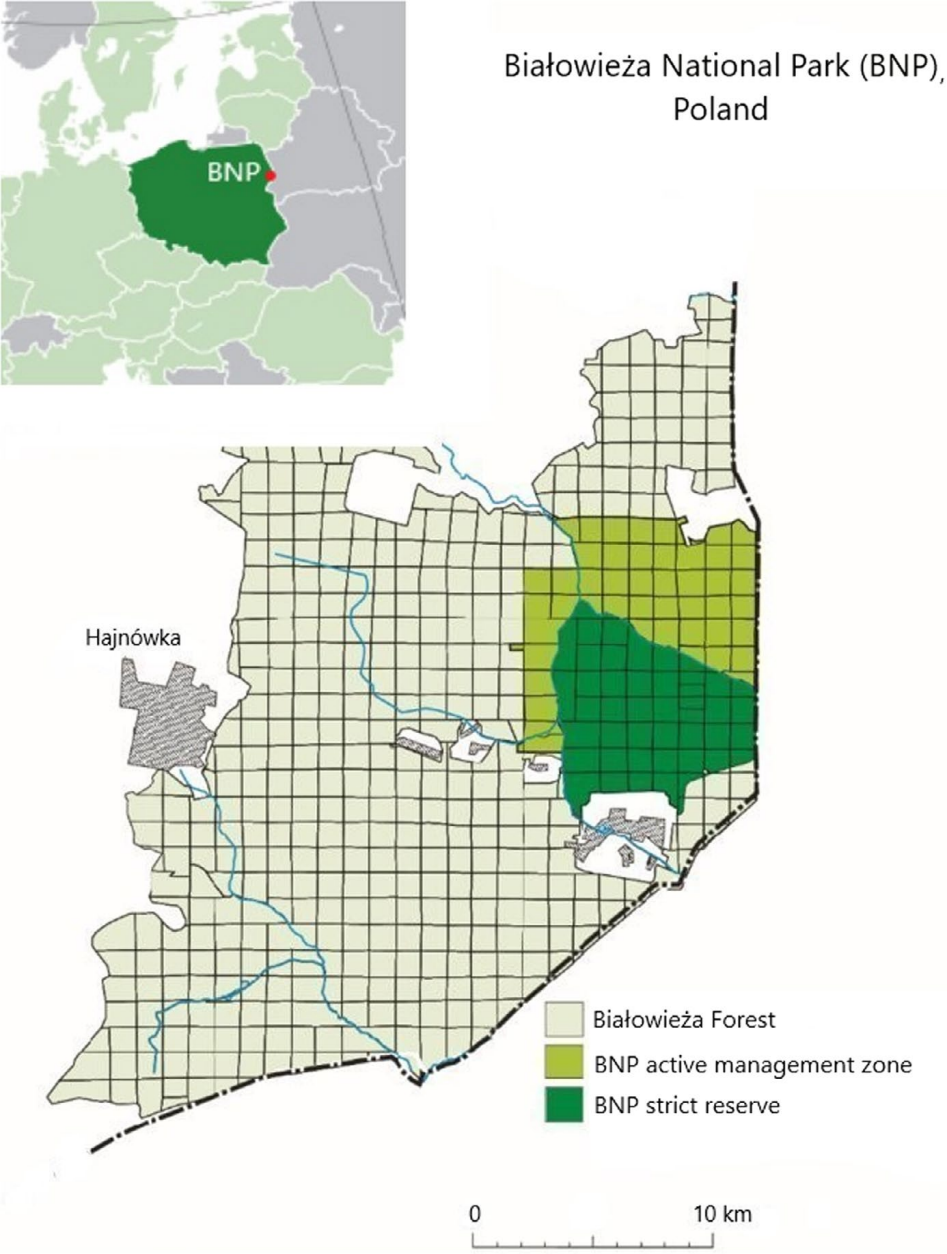
Locality of the study area (in the context of Poland and Białowieża Forest)

To gain an insight into the colonization process of fruiting bodies of bracket fungi by invertebrates, it was decided to select Białowieża National Park (BNP) in eastern Poland as the study area. This selection was not an arbitrary one, since the BNP is considered to be the largest area of natural forest in the North European Plain (Gutowski & Jaroszewicz, 2004). Therefore, the BNP is widely recognized as a model forest, to which various observations and research of natural deciduous and mixed forests in this climate zone can be referred (Gutowski, 2004). Białowieża National Park is the oldest Polish national park (established in 1947). It is located in the northeastern part of Poland (52°69′89′′ N–52°81′89′′ N, 23°71′76′′ E–23°93′95′′ E) (Figure 1). The maximum altitude in the BNP is 176.3 m above sea level. According to Sokołowski (2004), in the Białowieża Primeval Forest, inside which the Białowieża National Park is located, there are 27 forest communities with 24 species of trees. BNP is covered mainly by the East‐European oak*–*hornbeam forest *Tilio–Carpinetum typicum*, *stachyetosum*, *caricetosum*, *circaeaetosum,* and *calamagrostietosum* (48.7% of the BNP’s area). The valleys of the primeval forest watercourses, which are periodically flooded, are covered with an ash‐alder riparian forest *Circaeo–Alnetum* (8.35%), whereas in the peatland valleys, marshy interior basins and alder swamp forests *Ribeso nigri–Alnetum* and *Sphagno squarrosi–Alnetum* (5.86%) can be found. The boreal spruce forest growing on peatland *Sphagno girgensohnii–Piceetum* (1.59%) and sub‐boreal swampy birch forest *Thelypteridi–Betuletum pubescentis* (5.26%) are other plant associations that can be found there. Small phytocenoses of the poorest habitats growing on sands are covered with pine forests, primarily subcontinental fresh coniferous forest *Vaccinio vitis–idaeae–Pinetum* and midland dry pine forests *Cladonio–Pinetum* (3.80%), while swampy coniferous forest *Vaccinio uliginosi–Pinetum* and raised‐bog community *Ledo–Sphagnetum magellanici* (1.69%) grow on a shallow peatland (Jaroszewicz, 2010; Kwiatkowski & Gajko, 2009). Both bracket fungi (Domański, 1964, 1967; Niemelä, 2013) and numerous invertebrate groups (e.g., Gutowski & Krzysztofiak, 1995; Jaworski et al., 2014; Stańska et al., 2016) have already been researched at Białowieża primeval forest, which facilitates a comparison of the obtained results with the current knowledge on the subject matter. It is worth noting that this research is the first one on the subject of invertebrate assemblages inhabiting fruiting bodies of bracket fungi in the BNP area.

### 2.2 Collecting material

In the course of a survey of bracket fungi conducted in the years 2008–2012, a total of 142 species were recorded at BNP (Niemelä, 2013). However, during the fieldworks for this manuscript only the most numerously represented species were collected. In the months from June to August in 2014, a total of 100 specimens (fruiting bodies) belonging to 10 species of bracket fungi were harvested. For that purpose, an axe was used to cut the bracket fungi off of tree trunks.

### 2.3 Laboratory procedures

The fruiting bodies were identified at the laboratory, and their DD was determined (Table 1). The classification of the DDs of fruiting bodies had not been used so far—it is proposed by the authors, and like the wood decay scales (e.g., Maser et al., 1979), it is based on the differences in the occurrence of various features in the substrate with a different degree of decay. Next, in order to extract mesofauna, the fruiting bodies were placed in Tullgren funnels for 72 hr.

**TABLE 1:** Decay scale of bracket fungi. DD—degree of decay

DD	Description
1.	Fruiting body with fresh hymenophore, without visible signs of decay.
2.	Fruiting body with dry hymenophore, without visible signs of decay.
3.	Fruiting body with few traces of decomposition, for example, single (up to 10) insect galleries.
4.	Fruiting body with numerous traces of decay, such as insect galleries, easily crumbled.

The collected invertebrates were preserved in 75% ethanol and then classified into seven taxonomic groups: spiders (Aranae), Opiliones, pseudoscorpions (Pseudoscorpionida), two groups of mites (Mesostigmata, Oribatida), springtails (Collembola), and insects (Insecta). Due to the fact that there was only one species of Opiliones in the research, and its trends were very similar to that of spiders, Aranae and Opiliones were connected into one group; therefore, in the further part of the article, six groups are mentioned, and spiders and Opiliones are analyzed and discussed together.

The collected Araneae and Opiliones were identified and counted. The taxonomic keys of valid spiders and Opiliones (Nentwig et al., 2020; Roberts, 1985; Rozwałka, 2017) were used to identify the species.

The collected individuals of Pseudoscorpionida were examined using a stereomicroscope. For some species, it was necessary to prepare temporary microscope slides so that they could be studied under the compound microscope. The taxonomic keys of Beier (1963) and Christophoryová et al., (2011), Christophoryová Šťáhlavský, and Fedor (2011) were used to identify the species.

In order to identify Mesostigmata, both semi‐permanent (using lactic acid) and permanent (using Hoyer's medium) microslides were prepared. All the individual mesostigmatic mites were examined using a light microscope (Zeiss Axioskop 2) and taxonomical literature (e.g., Gwiazdowicz, 2007, 2010; Karg, 1993; Mašán, 2001).

The Oribatida were identified using a light microscope, preferably with a phase contrast and a differential interference contrast. Prior to the examination, the cuticles were rendered transparent using concentrated lactic acid, 60% lactic acid, or lactophenol. The clearing process was performed at room temperature over a course of several days, and sometimes weeks. Oribatid mites were determined at a species level by identifying their key features and original species descriptions (Niedbała, 2008; Olszanowski, 1996; Weigmann, 2006). The names of the species were confirmed according to Weigmann (2006).

The Collembola were identified using a light microscope. The extracted specimens of springtails were mounted with Hoyer's medium (Coleman et al., 2004) to prepare the permanent microscopic slides necessary for the taxonomic analysis. The taxonomic identification of the Collembola was carried out by following the manuals of Fjellberg (1998, 2007), Bretfeld (1999), Potapov (2001), Thibaud et al., (2004), Dunger and Schlitt (2011), and Jordana (2012).

The identification of immature stages of collected Insecta was carried out by following Stehr's manuals (Stehr, 2005; Stehr, 2008). Imagines of Coleoptera were counted and identified using taxonomic descriptions (e.g., Burakowski & Ślipiński, 1986; Stebnicka, 1991; Szymczakowski, 1961) and a comparative collection from the Natural History Collections, AMU.

Here, invertebrates analyzed in this study were identified as higher taxonomic units, for example, order or family, and were included in the statistical analyses as separate species.

The specimens collected for this work were deposited in the acarological collection at Adam Mickiewicz University, Faculty of Biology, the Natural History Collections, Poznań, Poland (Araneae and Opiliones, Pseudoscorpionida, Insecta), the Department of Forest Pathology, Poznań University of Life Sciences (Mesostigmata), the University of Silesia, Poland (Oribatida), and the Cardinal Stefan Wyszyński University, Poland (Collembola).

### 2.4 Statistics

In order to assess the differences between the samples in terms of the invertebrate composition to avoid interpretation problems related to data compression, the detrended correspondence analysis (DCA) developed by Hill and Gauch (1980) was used. Such problems typically occur while adopting simple correspondence analysis and burdening the data with gradient. Moreover, the permutational multivariate analysis of variance (MANOVA) was used to verify the hypothesis on the equality of bracket fungi in terms of the number of species and individuals colonizing them. The numbers of individuals in each species were the dependent variables, and DDs were factor levels. Since the range of values for the analyzed data was vast, the data had been transformed by applying Wisconsin double standardization prior to the analyses (the data were square‐root‐transformed and then subjected to the Wisconsin double standardization, where the species are first standardized by maxima and then each site divided by site total). In order to present the differing groups corresponding to their individual DDs (for summarized and Wisconsin standardized data in each DDs), the methods used were the cluster analysis with Manhattan distance and Ward's method (Maechler et al., 2019). The invertebrates significantly influencing the variation in DDs were identified using the indicator species analysis (De Cáceres et al., 2010). All of the statistical analyses were conducted in the R environment following the procedures from the vegan package (Oksanen et al., 2019) and the indicspecies package (De Cáceres & Legendre, 2009).

## 3 RESULTS

### 3.1 General information

The number of bracket fungi varied within each species, which was a result of the biology and ecology of these fungi and was also due to their more numerous or sporadic occurrence. The following 10 species were collected: *Fomitopsis pinicola* (47 specimens), *Fomes fomentarius* (18), *Ganoderma applanatum* (11), *Porodaedalea pini* (9), *Trametes versicolor* (4), *Gloeophyllum odoratum* (3), unidentified (3), *Stereum hirsutum* (2), *Hymenochaete rubiginosa* (1), *Stereum rugosum* (1), and *Trametes gibbosa* (1). The number of fruiting bodies within the individual DDs was comparable at 1 DD—23, 2 DD—24, 3 DD—23, and 4 DD—30.

The number of individuals collected per one sample (one bracket fungus) ranged from 0 to 1,385 (mean: 160.69 ± 24.64), while the number of species collected per one sample ranged from 0 to 46 (mean: 12.25 ± 0.85). The total number of collected invertebrates was 16 068, and the number of species was 224. In terms of the number of species, the richest groups were mites: Oribatida (115 species) and Mesostigmata (52), followed by Collembola (19), Insecta (26), Aranae, and Opiliones (9), whereas Pseudoscorpionida were represented by the lowest number of taxa (3). In terms of the number of individuals, the most abundant were Oribatida mites (12 543 individuals), followed by insects (1695), Mesostigmata mites (1,421), Collembola (319), and Pseudoscorpionida (70), while the least numerous were Aranae and Opiliones (20). *Carabodes femoralis* (8,811 individuals), *Carabodes subarcticus* (1889), and *Cis* spp. (1,166) were the most numerous species (Appendix ).

### 3.2 Diversity of invertebrate assemblages depending on DD

**FIGURE 2:**
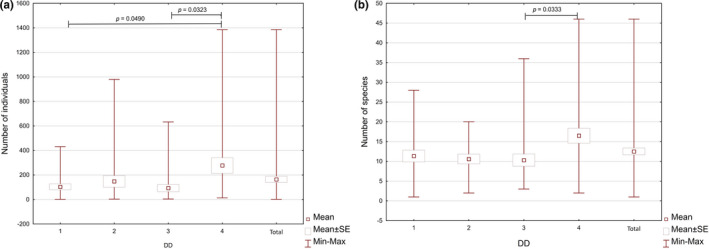
Minimum, maximum, mean number ± SE of individuals (a) and species (b) of invertebrates depending on DD of samples (1‐4—degrees of decay of fungi)

The study assumed that both the number and the species’ richness of invertebrates depend on the DD of bracket fungi. In order to verify this thesis, the data analyses were conducted in order to indicate a trend for the occurrence of a higher mean number of individuals and species per sample in the higher DD classes (Figure 2a,b); however, this trend appeared to be nonlinear. Although in all of the analyzed invertebrate groups, the mean number of species and individuals per sample in the highest DD class was higher than in 1 DD. The species variation and the number of individual invertebrate groups in the samples, belonging to two intermediate DD, differed (Figure 2a,b). Only in the case of Pseudoscorpionida and Insecta, the mean number of species per sample in 2 DD was lower than in 3 DD, whereas only in Pseudoscorpionida, Collembola, and Insecta the mean number of individuals per sample in the 2 DD group was lower than in the 3 DD group (Figures 5b,e,f and 6b,f).

While analyzing the obtained results, it may also be observed that certain invertebrate species, such as *Eniochthonius minutissimus*, were present in all DD classes, except 3 DD. There were also some others, which were detected both in fresh fruiting bodies and in those with the highest degree of decay, in contrast to the intermediate DD, for example, spiders from the family Linyphiidae, *Lophopilio palpinalis,* or *Caleremaeus monilipes*. However, selectivity was most frequently observed in a specific DD of bracket fungi. Examples of species preferring fresh fruiting bodies with no signs of decay include mites such as *Hoploseius mariae*, *Hoploseius oblongus,* or *Dissorhina ornata,* while in bracket fungi in the 4 DD mite species such as *Dendrolaelaps pini* (mesostigmatic mites were most numerously represented) or *Carabodes femoralis* (the most numerous species recorded in this study) were more frequent. It was less frequent for a given species to be reported in the greatest numbers in bracket fungi with 2 DD and 3 DD. Such an example may be provided by the oribatid mites such as *Carabodes coriaceus*, *Chamobates cuspidatus,* and *Neoribates aurantiacus*.

**FIGURE 3:**
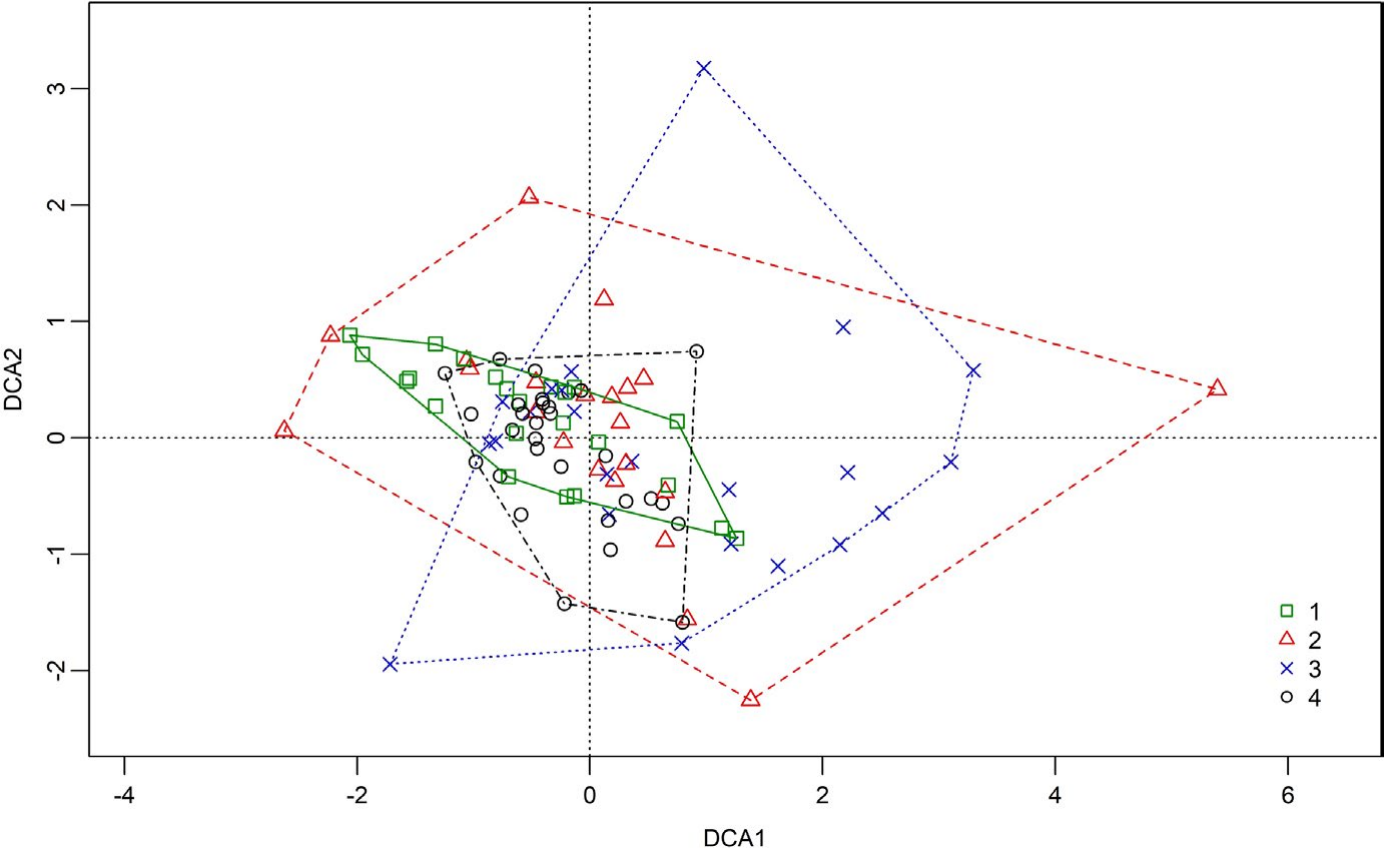
Detrended correspondence analysis (DCA) of samples based on the Bray–Curtis dissimilarities of double standardization abundances with clusters for groups of samples. 1‐4—samples belonging to particular DDs

In order to determine whether DD has effect on the number of individuals and species of invertebrates colonizing bracket fungi, the DCA was conducted (using the decorana function in the R platform). The results of this analysis are presented in Figure 3. The first two axes indicate 54.86% species variation, with the first axis accounting for 30.35% and the second axis for 24.52%. It is evident that most points corresponding to the individual samples form one cluster. The application of DCA made it possible to “expand” this cluster, thanks to which a division of samples into groups in terms of their DD may be observed. Thus, the analysis provided a partial grouping of bracket fungi in terms of their DDs and indicated similarities between these groups. In the DCA, the groups are considered to be different if they are separated linearly. Figure 3 shows no marked divisions between groups; however, certain similarities may be observed.

The DCA and cluster analysis reveal a degree of differences between DDs. Thus, the permutation analysis of variance (adonis function) was conducted. The homogeneity criterion of multivariate dispersions is fulfilled (*p* = .731, *F* = 0.468; *df*: 3, 94, function permutest). It was shown that the effects of DDs differ significantly (*p = *.001, see Tab. 5), but since *R* ^2^ = 0.061 this suggests an even distribution of high and low ranks, both within and between groups. In addition, only 6% of the variation in distance between the groups is explained by grouping testing. As a result, the groups differ significantly, but these differences are not distinctly evident.

**FIGURE 4:**
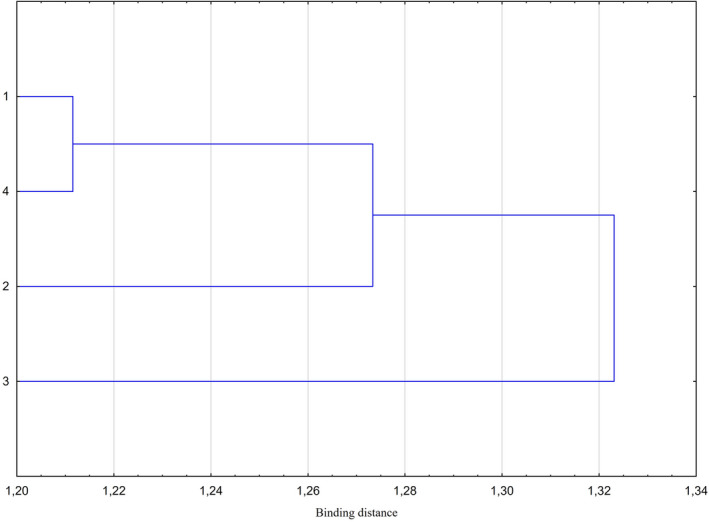
Cluster (Ward method) analysis of DD groups based on the Bray–Curtis dissimilarities of double standardization sum of abundances. 1‐4—DDs

The result obtained in the discussed analysis is consistent with the results of cluster analysis (Figure 4). This shows that samples belonging to 1 DD and 4 DD form one cluster (in Figure 3 mixed points), continuing on 2 DD can also be added to this cluster, and lastly 3 DD was also added, but it may also be considered a separate cluster.

Moreover, the pairwise comparisons were conducted using a permutation analysis of variances method (adonis function) with the Bonferroni correction of *p*‐values (Table 2). It was shown that the mean numbers of invertebrates on bracket fungi with 1 DD differ significantly from the mean number of invertebrates on bracket fungi with 3 DD and 4 DD. At the same time, the mean numbers of invertebrates in 2 DD samples differ significantly from the mean number of invertebrates in 4 DD samples. This result differs from the result provided by the cluster analysis. This may have been caused by the fact that there are no distinct differences between DDs (see Figure 3), which is also confirmed by a low value of *R* ^2^ with the permutational MANOVA.

**TABLE 2:** Permutation MANOVA and *p*‐value of pairwise comparison on the Bray–Curtis distances for double Wisconsin standardization abundances (1,000 permutation, adonis function)

Effects	Df	SS	MS	Pseudo*F*	*p*
Degree of decay	3	1.961	0.6536	2.041	0.001
Residuals	94	30.103	0.3202		Z
Total	97	32.064			
Degree of decay	1	2	3		
2	0.863				
3	0.012	0.060			
4	0.006	0.024	0.114		

The conclusions based on DCA, the cluster analysis, and permutational MANOVA are ambiguous, which is why in the DCA graph the areas identified by individual DDs have been marked (Figure 3). No area is disjointed from the others; however, it can be seen that the areas of 1 DD and 4 DD are clustered the most and as a result are also most similar. As far as 2 DD and 3 DD are concerned, they are the most scattered ones; the greatest variability in the number and population size of invertebrates has been found in the samples belonging to the 2 DD group and the smallest for 4 DD. Along DCA2, the greatest variability was observed for the 3 DD samples. The means along DCA1 indicate a gradient of 1 DD (−0.507953), 4 DD (−0.231116), 2 DD (0.099782), and 3 DD (0.713966), means for 1 DD and 4 DD are found at one end and are similar, whereas 3 DD is on the other one with 2 DD in the center. This confirms the assumptions provided by cluster analysis. The means along DCA2 (0.1623248, 0.1030077, −0.1136265, and −0.1128757; form 1 DD to 4 DD) indicate the gradient: 3 DD, 4 DD, 2 DD, and 1 DD.

Thus, 3 DD and 4 DD are similar analogously to 2 DD and 1 DD, which confirms the result obtained from permutational MANOVA. The largest areas cover the 2 DD and 3 DD samples, as a result, it may be concluded that these samples are most abundant in terms of the number of invertebrate individuals and species.

The indicator species analysis (using the multipatt function from the indicspecies package), performed in order to show which invertebrates contributed to the group differentiation, indicates 11 taxa to distinguish the groups. There are 10 taxa associated with 1 group and 1 species associated with 2 groups (Table 3). A characteristic feature for the 1 DD samples is connected with the occurrence of invertebrates of Coleoptera, *Hoploseius oblongus*, *Dissorhina ornata,* and *Pseudisotoma sensibilis*, while for 2 DD samples—*Orchesella bifasciata*. The 3 DD samples encompass *Chernes cimicoides*, *Scheloribates latipes,* and *Gamasellodes bicolor*, and those with 4 DD—*Dinychus perforates* and Elateridae (Table 3). *Lasioseius muricatus* contributed to the homogeneity of 1 DD and 2 DD. This analysis shows that there are a limited number of invertebrates, which are characteristic for each DDs (the greatest number for 1 DD). It is also worth mentioning that many invertebrate species in this study are common for most DDs, while many other species were only represented by one individual in one sample.

**TABLE 3:** Species characteristic for a group of sites, detected using indicator species analysis

Groups	Species	Stat	*p* value
1	Coleoptera	0.348	0.005
	*Hoploseius oblongus*	0.296	0.016
	*Dissorhina ornata*	0.272	0.031
	*Pseudisotoma sensibilis*	0.267	0.041
2	*Orchesella bifasciata*	0.237	0.044
3	*Chernes cimicoides*	0.321	0.005
	*Scheloribates latipes*	0.278	0.02
	*Gamasellodes bicolor*	0.26	0.036
4	*Dinychus perforates*	0.335	0.009
	Elateridae	0.321	0.007
1 + 2	*Lasioseius muricatus*	0.275	0.019

### 3.3 The effect of DD fruiting bodies on individual invertebrate groups

**FIGURE 5:**
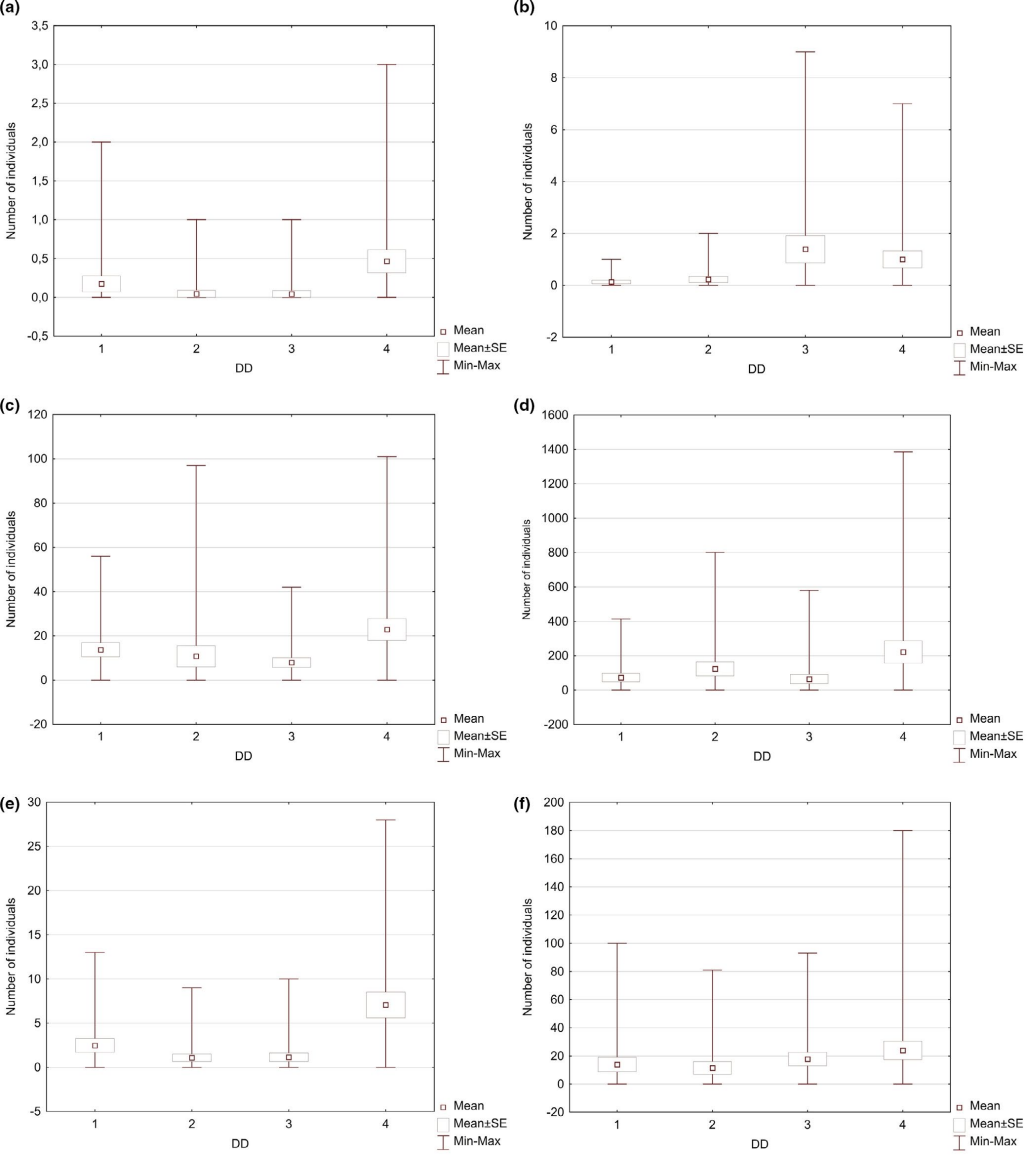
Minimum, maximum, mean number ± SE of individuals of invertebrates (a—Aranae and Opiliones, b—Pseudoscorpionida, c—Acari, Mesostigmata, d—Acari, Oribatida, e—Collembola, f—Insecta) depending on DD of samples (1‐4—degrees of decay of fungi)

**FIGURE 6:**
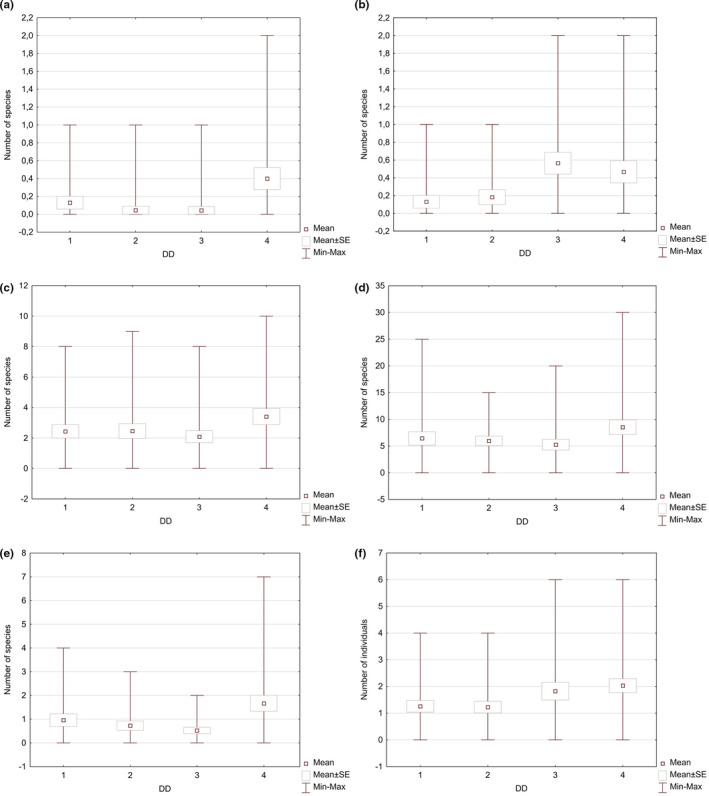
Minimum, maximum, mean number ± SE of species of invertebrates (a—Aranae and Opiliones, b—Pseudoscorpionida, c—Acari, Mesostigmata, d—Acari, Oribatida, e—Collembola, f—Insecta) depending on DD of samples (1‐4—degrees of decay of fungi)

While analyzing the obtained results, it was also observed that the individual invertebrate groups react differently to the changes in the DD of the substrate. In the case of spiders and Opiliones in bracket fungi of the 1 DD group, three species were reported and were represented by four individuals (these species were also recorded in the 4 DD bracket fungi). In turn, bracket fungi with 2 DD were colonized by only one individual from the Lycosidae family (also recorded in 4 DD bracket fungi). In the case of bracket fungi with 3 DD, one species was reported (*Theridion* sp.), which was not found in the other DD groups. The bracket fungi with 4 DD were the richest in terms of both the number of species and the number of individuals. Among the fungi, 8 species were identified and represented by a total of 14 individuals, while other species such as *Helophora insignis*, *Pirata hygrophilus,* or *Thyreosthenius parasiticus* were reported only in this DD group (Figures 5a and 6a, Appendix ).

The results concerning Pseudoscorpionida were slightly different. In the 1 DD group, three individuals from the Chernetidae family were identified. The same species was also found in the samples of all DD groups. In the 2 DD samples, two species were reported (5 individuals in total), while both 3 DD and 4 DD reported three species of pseudoscorpions each. In addition, the number of individuals was also similar, with 32 and 30 individuals, respectively (Figures 5b and 6b).

As far as Mesostigmata mites in the 1 DD samples are concerned, a total of 23 species and 315 individuals were reported. Some of them, such as *Ameroseius corniculus*, *Ameroseius longitrichus*, *Dendrolaelaps arvicolis,* or *Hoploseius mariae*, were found solely in the samples of that particular DD. *Hoploseius oblongus* on the other hand was recorded in this DD group much more frequently than in the other groups. In fruiting bodies classified as 2 DD group, a total of 237 individuals of Mesostigmata mites were recorded, belonging to 26 species. Some of them, such as *Ameroseius imparsetosus*, *Holoparasitus calcaratus*, *Sejus polonicus*, or *Zerconopsis michaeli*, were reported only in the samples from that particular DD group, while *Dendrolaelaps cornutulus*, *Zerconopsis decemremiger*, and *Zerconopsis remiger* were reported in 2 DD group in greater numbers than in the samples from other DD groups. In the 3 DD samples, a total of 183 individuals and 24 Mesostigmata mite species were recorded. The species such as *Dendrolaelaps trapezoides*, *Epicriopsis horridus*, *Iphidonopsis dendrophilus*, *Ololaelaps placentula*, *Parazercon radiatus*, and *Proctolaelaps fisheri* were identified only in this DD group, while *Gamasellodes bicolor* or *Uroobovella vinicolora* were found in the 3 DD samples much more frequently than in those belonging to the other DD groups. In the 4 DD samples, both the largest number of species (32) and the highest number of individuals (686) of Mesostigmata mites were reported (Figures 5c and 6c). Nine species, for example, *Dendrolaelaps stammeri*, *Dendrolaelaps tenuipilus*, or *Geholaspis longispinosus,* were found only in the samples from this DD group, whereas species such as *Dendrolaelaps pini*, *Dendrolaelaps punctatulus,* or *Dinychus perforatus* were found in the 4 DD samples in larger numbers than in the other DD groups.

Oribatida mites were the most numerous invertebrate group in terms of both the number of species and the number of individuals in the analyzed material. In fruiting bodies of 1 DD, a total of 1677 individuals belonging to 58 species were recorded. Some of them, for example, *Carabodes tenuis*, *Cepheus dentatus,* or *Chamobates pusillus*, were found only in the samples from this DD group, while *Carabodes areolatus*, *Dissorhina ornata,* or *Melanozetes mollicomus* were observed in the samples from this DD group in much greater numbers than in the other groups. In the 2 DD samples, a total of 2,732 individuals were identified as belonging to 47 species. Some of them, such as *Adoristes ovatus*, *Steganacarus* (*Atropacarus*) *striculus*, or *Damaeus crispatus*, were reported only in the samples of this DD group. In turn, species such as *Carabodes coriaceus*, *Carabodes labyrinthicus*, or *Chamobates cuspidatus* were observed in the fruiting bodies of 2 DD in greater numbers than in the other DD groups. In the 3 DD samples, the lowest number of individuals was detected (1,477); however, the number of species was slightly higher than in the fruiting bodies belonging to the 2 DD group, which was 51. Some species, for example, *Conchogneta dalecarlica*, *Spatiodamaeus fageti,* or *Eremaeus tuberosus,* were observed solely in the samples from this DD group, while *Damaeus* (*Paradamaeus*) *clavipes*, *Spatiodamaeus boreus,* or *Zygoribatula exilis* were found in the samples of this DD group in greater numbers than in the other groups. In the fruiting bodies from the 4 DD group, both the highest number of individuals (6,653) and the greatest number of species (88) of Oribatida mites were recorded (Figures 5d and 6d). It needs to be emphasized that 26 species, for example, *Achipteria nitens*, *Conchogneta traegardhi,* and *Cultroribula bicultrata*, were detected only in the 4 DD samples, while other species, for example, *Carabodes femoralis*, *Hypochthonius rufulus,* and *Nanhermannia nana,* were observed in the 4 DD fruiting bodies more frequently than in the other samples.

In all of the samples, a total of 319 individuals and 19 species of Collembola were reported. There were 57 individuals belonging to 9 species found in the fruiting bodies with 1 DD. Two species (*Pseudisotoma sensibilis* and *Sinella myrmecophila*) were recorded in the samples from this DD group more frequently than in the fruiting bodies from the other DD groups. In the 2 DD samples, 24 individuals belonging to 8 species were found. *Orchesella bifasciata* was the only species recorded solely in the 2 DD samples, while *Pseudachorutella assigilata* and *Pseudosinella immaculata* were found in bracket fungi from these DD groups in greater numbers than in the other groups. In the 3 DD samples, 26 individuals representing 7 species were recorded. *Entomobrya multifasciata* was the only species found solely in the 3 DD samples. Springtails were most numerously represented in terms of both individuals (212) and species (14) in the 4 DD samples (Figures 5e and 6e). Six species were reported, among them *Arrhopalites coecus* and *Cyphoderus albinus*, and found only in this DD group, while *Caprainea marginata*, *Entomobrya corticalis,* and *Tomocerus minor* were recorded in the 4 DD samples more frequently than in the other groups.

The last group of invertebrates identified in this study comprised of insects. In the 1 DD samples, a total of 320 individuals were detected belonging to 8 species. *Cyphon* sp. was the only species of insects recorded solely in the 1 DD samples, while Coleoptera and Diptera were found in bracket fungi samples with this DD more numerously than in the other groups. In the 2 DD samples, more individuals (251) and a larger number of species (11) of insects were recorded. *Nargus velox*, Dermaptera and Psocoptera, were found only in the fruiting bodies of the DD group, while Thysanoptera were identified in the 2 DD samples more frequently than in the other groups. In the 3 DD samples, even greater numbers of individuals (407) and species (16) of insects were found. Only in the fruiting bodies of this DD group did the analyses show the presence of *Attagenus* sp., *Bolitophagus reticulatus*, *Corticaria* sp., or Dermestidae, whereas *Ennearthron* sp., *Octotemnus* sp., and Ciidae in the 3 DD samples were found more frequently than in the other groups. In the 4 DD samples, the presence of 717 individuals belonging to 13 insect species was reported (Figures 5f and 6f). Five of them, for example, *Acrotrichis* sp., *Cerylon fagi,* or *Cerylon ferrugineum,* were found solely in the samples from this DD group, whereas *Cis* spp. were markedly more numerous in the most decayed samples compared with the other DD groups.

## 4 DISCUSSION AND CONCLUSIONS

Numerous studies have shown that bracket fungi inhabit specific habitats, described as patchy (often ephemeral), resembling an extremely heterogeneous environmental mosaic rather than "islands" in the true sense of the word (e.g., O'Connell & Bolger, 1997a, 1997b). Sporocarps of polypore fungi represent well‐defined, patchy, and temporary habitats, which have long‐fascinated ecologists and biodiversity researchers (Hågvar & Steen, 2013). They may be termed biological hotspots, supporting high numbers of species within small volumes (Komonen, 2003). The evidence for the unique character of this type of microhabitat may be provided, for instance, by the occurrence of organisms reported practically only in such habitats, as it is the case with such insect species as, for example, *Megaselia lobatafurcae* and *M. parspallida* (Disney & Pagola‐Carte, 2009), or certain mite species, for example, from the *Hoploseius* genus (e.g., Gwiazdowicz, 2002; Mašán & Halliday, 2016; Walter, 1998). It also needs to be emphasized here that some invertebrate taxa, such as *Hoploseius tenuis*, even indicate certain morphological adaptations (an elongated and narrow body) to live within the pores of bracket fungi (Lindquist, 1965, 1995).

Some species reported in this microhabitat are also found in other similar habitats, such as feeding galleries of insects located under bark or in decaying wood (e.g., Salmane & Brumelis, 2010). The invertebrate species found in the greatest numbers in this study were also reported in microhabitats other than fungal fruiting bodies. *Carabodes femoralis*, a mycophagous, decomposer oribatid mite (Manu & Honciuc, 2010; Schneider et al., 2005), is the most numerous species among all the invertebrates present in this study. It is found mainly in litter and soil (e.g., Błoszyk & Olszanowski, 1997; Manu & Honciuc, 2010; Seniczak et al., 2006), as well as in the nests of *Formica rufa* ants (Sell, 1990), cave mud, deadwood, leaves, and guano (Maślak & Barczyk, 2011). In addition, the invertebrate species ranking second in the terms of its numbers, *Carabodes subarcticus*, was also found in such microhabitats as soil (e.g., Hågvar et al., 2014; Kagainis, 2010, 2015) and bark of deadwood (Bluhm et al., 2015). In the case of the most numerously found invertebrate taxa from the other groups, it may also be observed that apart from fungal fruiting bodies they are also observed in other substrates. Spiders from the Linyphiidae family are also found in various habitats, with only a slight preference to forests (Wiśniewski et al., 2018), agricultural habitats (Downie et al., 2000; Schmidt & Tscharntke, 2005), and grass (Thomas & Jepson, 2003). *Chernes cimicoides*, the pseudoscorpion taxon most frequently recorded in this study, was also identified in tree hollows under tree bark (Krajčovičová & Christophoryová, 2014), under deadwood bark (Krajčovičová et al., 2018), in window traps at the bark and trunk eclectors (Muster & Blick, 2015), with an indigenous species found in bird nests (Christophoryová, Krumpálová, et al., 2011; Christophoryová, Šťáhlavský, et al., 2011). The Mesostigmata mite recorded in greatest numbers in this study, *Dendrolaelaps pini*, was also found in decayed wood (Gwiazdowicz et al., 2011), *Ips typographus* galleries (Salavatulin et al., 2018), pine stumps and under wing covers of *Hylurgus ligniperda* and *Hylastes* sp. (Hirschmann & Wiśniewski, 1982). *Entomobrya corticalis*, the most numerous among the identified springtail species, is also known to live in such microhabitats as deadwood, epiphytic mosses, litter, and soil (Daghighi et al., 2013; Skarżyński et al., 2016; Yahyapour et al., 2019). Also, less numerous species, such as *Pseudisotoma sensibilis* and *Orchesella bifasciata,* are mentioned as corticolous and bryophilous species (Fjellberg, 2007; Weiner, 1981). Among Coleoptera found in this study, the most numerous were members of the Ciidae family—beetles developing entirely inside fungal bodies (Graf‐Peters et al., 2011). Also found were beetles which are not directly connected with the bracket fungi, for example, necrophagous Dermestidae and *Nargus velox*—the species representative of the family Cholevinae, sporadically observed in Poland (Jałoszyński et al., 2006), as well as those typically found under the tree bark (e.g., *Cerylon* spp.). The presence of Coleoptera is considered clearly as accidental in this type of microhabitat (e.g., *Cyphon* sp.) and can be explained by the search for a temporary shelter.

While there are no publications covering directly the subject of changes in the structure of invertebrate fauna colonizing fungal fruiting bodies depending on the degree of decay, examples may also be provided from a slightly similar, but a better‐known substrate, that is, the decaying wood. The results of the research conducted by Braccia and Batzer (2001), Skubała and Sokołowska (2006), or Gwiazdowicz et al., (2011) show that with the progressing decay of the substrate, the number of invertebrate species colonizing it generally increases. Similarly, as it has been observed for the number of exclusive species, although in the latter case this increase is not linear. The results of this study indicate a similar dependence; additionally, they also reveal that various groups of invertebrates react differently to the progressing decay of the substrate. The differences between the invertebrate assemblages colonizing the samples belonging to the individual DD groups were clearly demonstrated by the results of the DCA, indicating that the samples with 2 DD and 4 DD are very similar to one another in terms of invertebrate assemblages, which may be justified by the fact that most observations are contained within a similar area (only single observations of group 2 DD are scattered outside the common distribution area). The observations of 3 DD differ the most from the other DDs. The set of these observations is shifted most to the right in relation to the other DDs. A study conducted by Gwiazdowicz et al., (2011) also suggested that certain mite species are more associated with a specific but not necessarily high DD of the substrate, while in the other DD groups they are found in fewer numbers. Also, in the case of this study, despite the analysis of the slight substrate differences, similar dependencies have been observed—this was shown by means of indicator species analysis, used to identify the invertebrate species, which distinguished individual DD classes. This analysis showed not only which specific invertebrate species distinguish individual DD groups, but also the fact that the number of these species is small, since a considerable proportion of the invertebrate species colonized samples from several DD groups.

Summing up, the results have confirmed the hypothesis concerning the diversification of invertebrate assemblages colonizing bracket fungi depending on the degree of their decay. Similarly, as in the case studies concerning the fauna colonizing decaying wood, it has also been observed that the largest number of individuals and invertebrate species of all taxonomic groups, with the exception of Pseudoscorpionida, was recorded in the most decayed samples. An increase in the population size and diversity of invertebrates with the progressing decay of the substrate was not linear in this study, and it varied between individual groups of organisms. In the case of spiders and Opiliones, Mesostigmata mites, and springtails, the lowest numbers of species and individuals were observed in the samples from the two intermediate DD groups. The figures were slightly higher in the least decayed samples and the highest in the most decayed fruiting bodies. In turn, in Pseudoscorpionida the lowest number of species and individuals was found in the least decayed samples, while they were highest in the 3 DD group. The lowest number of species and individuals of Oribatida mites was recorded in the 3 DD samples, whereas the highest numbers of both species and individuals of this group—in the 4 DD samples. In the case of insects, the lowest number of individuals was recorded in the 3 DD samples, while the number was highest in the most decayed fruiting bodies. In turn, the lowest number of taxa from this invertebrate group was observed in the 2 DD samples and the highest number—in fruiting bodies with the highest DD. The problem of decomposition of fungal fruiting bodies and the structural and chemical changes that take place at that time, as well as the assemblages of invertebrates occurring in fruiting bodies of varying degrees of decomposition, may be the subject of further research.

The results of this study show that fruiting bodies of bracket fungi constitute a microhabitat colonized by unique invertebrate assemblages, among which certain taxa are found almost solely in this habitat type. It also appears that the DD of fruiting bodies can play role in terms of biodiversity modification of colonizing invertebrates. This information may be useful both as supplementary data concerning the currently little‐known problems of invertebrate ecology. The data also entail sustainable forestry based on ecological principles, in which bracket fungi are treated not only as an indicator of potential economic losses, but also as an element of the natural environment having an impact on biodiversity.

## CONFLICT OF INTEREST

We have no conflicts of interest to disclose.

## AUTHOR CONTRIBUTIONS

**Anna Gdula:** Data curation (equal); Investigation (equal); Resources (equal); Writing‐original draft (equal). **Szymon Konwerski:** Investigation (equal); Methodology (equal). **Iza Olejniczak:** Investigation (equal); Methodology (equal). **Tomasz Rutkowski:** Investigation (equal); Methodology (equal). **Piotr Skubała:** Investigation (equal); Methodology (equal). **Bogna Zawieja:** Data curation (equal); Formal analysis (equal); Methodology (equal); Software (equal); Validation (equal). **Dariusz J. Gwiazdowicz:** Conceptualization (equal); Formal analysis (equal); Investigation (equal); Methodology (equal); Visualization (equal); Writing‐original draft (equal); Writing‐review & editing (equal).

## Supporting information

Supplementary Material

## Data Availability

Data are available from the Dryad Digital Repository (https://doi.org/10.5061/dryad.p5hqbzkpb).
