# Supplementary material for: The role of bracket fungi in creating alpha diversity of invertebrates in the Białowieża National Park, Poland
Source: Ecol Evol. 2021 Mar 31;11(11):6456–70. doi: 10.1002/ece3.7495 (PMC8207353; doi:10.1002/ece3.7495)
Supplement: Supplementary file 1 — Supplementary Material [file ECE3-11-6456-s001.doc]

|  |  | 1 DD |  |  | 2 DD |  |  | 3 DD |  |  | 4 DD |  |  | Total |  |
| --- | --- | --- | --- | --- | --- | --- | --- | --- | --- | --- | --- | --- | --- | --- | --- |
| Species | Abu | Fre | Dom | Abu | Fre | Dom | Abu | Fre | Dom | Abu | Fre | Dom | Abu | Fre | Dom |
| **Aranae and Opiliones** | 4 | 13.04 | 0.18 | 1 | 4.55 | 0.03 | 1 | 4.35 | 0.05 | 14 | 30.00 | 0.17 | 20 | 14.29 | 0.13 |
| *Amaurobius fenestralis* | 1 | 4.35 | 0.04 | 0 | 0.00 | 0.00 | 0 | 0.00 | 0.00 | 1 | 3.33 | 0.01 | 2 | 2.04 | 0.01 |
| Araneae | 0 | 0.00 | 0.00 | 0 | 0.00 | 0.00 | 0 | 0.00 | 0.00 | 2 | 3.33 | 0.02 | 2 | 1.02 | 0.01 |
| *Helophora insignis* | 0 | 0.00 | 0.00 | 0 | 0.00 | 0.00 | 0 | 0.00 | 0.00 | 1 | 3.33 | 0.01 | 1 | 1.02 | 0.01 |
| Linyphiidae | 2 | 4.35 | 0.09 | 0 | 0.00 | 0.00 | 0 | 0.00 | 0.00 | 4 | 13.33 | 0.05 | 6 | 5.10 | 0.04 |
| *Lophopilio palpinalis* | 1 | 4.35 | 0.04 | 0 | 0.00 | 0.00 | 0 | 0.00 | 0.00 | 2 | 6.67 | 0.02 | 3 | 3.06 | 0.02 |
| Lycosidae | 0 | 0.00 | 0.00 | 1 | 4.55 | 0.03 | 0 | 0.00 | 0.00 | 1 | 3.33 | 0.01 | 2 | 2.04 | 0.01 |
| *Pirata hygrophilus* | 0 | 0.00 | 0.00 | 0 | 0.00 | 0.00 | 0 | 0.00 | 0.00 | 1 | 3.33 | 0.01 | 1 | 1.02 | 0.01 |
| *Theridion* sp. | 0 | 0.00 | 0.00 | 0 | 0.00 | 0.00 | 1 | 4.35 | 0.05 | 0 | 0.00 | 0.00 | 1 | 1.02 | 0.01 |
| *Thyreosthenius parasiticus* | 0 | 0.00 | 0.00 | 0 | 0.00 | 0.00 | 0 | 0.00 | 0.00 | 2 | 3.33 | 0.02 | 2 | 1.02 | 0.01 |
| **Pseudoscorpionida** | 3 | 13.04 | 0.13 | 5 | 18.18 | 0.15 | 32 | 52.17 | 1.50 | 30 | 36.67 | 0.36 | 70 | 30.61 | 0.44 |
| *Chernes cimicoides* | 0 | 0.00 | 0.00 | 2 | 9.09 | 0.06 | 16 | 26.09 | 0.75 | 12 | 20.00 | 0.14 | 30 | 14.29 | 0.19 |
| Chernetidae | 3 | 13.04 | 0.13 | 3 | 9.09 | 0.09 | 11 | 26.09 | 0.52 | 12 | 13.33 | 0.14 | 29 | 15.31 | 0.18 |
| *Lamprochernes chyzeri* | 0 | 0.00 | 0.00 | 0 | 0.00 | 0.00 | 5 | 4.35 | 0.23 | 6 | 13.33 | 0.07 | 11 | 5.10 | 0.07 |
| **Acari, Mesostigmata** | 315 | 82.61 | 13.84 | 237 | 86.36 | 7.29 | 183 | 86.96 | 8.59 | 686 | 83.33 | 8.25 | 1421 | 84.69 | 8.90 |
| *Amblyseius* sp*.* | 0 | 0.00 | 0.00 | 0 | 0.00 | 0.00 | 1 | 4.35 | 0.05 | 1 | 3.33 | 0.01 | 2 | 2.04 | 0.01 |
| *Ameroseius corniculus* | 1 | 4.35 | 0.04 | 0 | 0.00 | 0.00 | 0 | 0.00 | 0.00 | 0 | 0.00 | 0.00 | 1 | 1.02 | 0.01 |
| *Ameroseius imparsetosus* | 0 | 0.00 | 0.00 | 1 | 4.55 | 0.03 | 0 | 0.00 | 0.00 | 0 | 0.00 | 0.00 | 1 | 1.02 | 0.01 |
| *Ameroseius longitrichus* | 4 | 8.70 | 0.18 | 0 | 0.00 | 0.00 | 0 | 0.00 | 0.00 | 0 | 0.00 | 0.00 | 4 | 2.04 | 0.03 |
| *Ameroseius* sp. | 0 | 0.00 | 0.00 | 4 | 4.55 | 0.12 | 0 | 0.00 | 0.00 | 0 | 0.00 | 0.00 | 4 | 1.02 | 0.03 |
| *Celaenopsis badius* | 3 | 4.35 | 0.13 | 1 | 4.55 | 0.03 | 2 | 8.70 | 0.09 | 4 | 10.00 | 0.05 | 10 | 7.14 | 0.06 |
| *Dendrolaelaps acornutus* | 0 | 0.00 | 0.00 | 0 | 0.00 | 0.00 | 13 | 13.04 | 0.61 | 19 | 6.67 | 0.23 | 32 | 5.10 | 0.20 |
| *Dendrolaelaps arvicolis* | 1 | 4.35 | 0.04 | 0 | 0.00 | 0.00 | 0 | 0.00 | 0.00 | 0 | 0.00 | 0.00 | 1 | 1.02 | 0.01 |
| *Dendrolaelaps cornutulus* | 3 | 8.70 | 0.13 | 9 | 9.09 | 0.28 | 0 | 0.00 | 0.00 | 0 | 0.00 | 0.00 | 12 | 4.08 | 0.08 |
| *Dendrolaelaps euarmatus* | 0 | 0.00 | 0.00 | 1 | 4.55 | 0.03 | 0 | 0.00 | 0.00 | 1 | 3.33 | 0.01 | 2 | 2.04 | 0.01 |
| *Dendrolaelaps pini* | 41 | 17.39 | 1.80 | 38 | 13.64 | 1.17 | 32 | 34.78 | 1.50 | 276 | 40.00 | 3.32 | 387 | 27.55 | 2.42 |
| *Dendrolaelaps procornutus* | 2 | 4.35 | 0.09 | 0 | 0.00 | 0.00 | 0 | 0.00 | 0.00 | 0 | 0.00 | 0.00 | 2 | 1.02 | 0.01 |
| *Dendrolaelaps punctatulus* | 1 | 4.35 | 0.04 | 1 | 4.55 | 0.03 | 0 | 0.00 | 0.00 | 36 | 6.67 | 0.43 | 38 | 4.08 | 0.24 |
| *Dendrolaelaps* sp. | 0 | 0.00 | 0.00 | 1 | 4.55 | 0.03 | 0 | 0.00 | 0.00 | 2 | 6.67 | 0.02 | 3 | 3.06 | 0.02 |
| *Dendrolaelaps stammeri* | 0 | 0.00 | 0.00 | 0 | 0.00 | 0.00 | 0 | 0.00 | 0.00 | 1 | 3.33 | 0.01 | 1 | 1.02 | 0.01 |
| *Dendrolaelaps tenuipilus* | 0 | 0.00 | 0.00 | 0 | 0.00 | 0.00 | 0 | 0.00 | 0.00 | 4 | 3.33 | 0.05 | 4 | 1.02 | 0.03 |
| *Dendrolaelaps trapezoides* | 0 | 0.00 | 0.00 | 0 | 0.00 | 0.00 | 3 | 8.70 | 0.14 | 0 | 0.00 | 0.00 | 3 | 2.04 | 0.02 |
| *Dendrolaelaps zwoelferi* | 1 | 4.35 | 0.04 | 2 | 9.09 | 0.06 | 13 | 4.35 | 0.61 | 12 | 6.67 | 0.14 | 28 | 6.12 | 0.18 |
| *Dinychus arcuatus* | 1 | 4.35 | 0.04 | 4 | 4.55 | 0.12 | 8 | 4.35 | 0.38 | 15 | 16.67 | 0.18 | 28 | 8.16 | 0.18 |
| *Dinychus perforatus* | 6 | 8.70 | 0.26 | 8 | 4.55 | 0.25 | 3 | 4.35 | 0.14 | 89 | 30.00 | 1.07 | 106 | 13.27 | 0.66 |
| *Epicriopsis horridus* | 0 | 0.00 | 0.00 | 0 | 0.00 | 0.00 | 1 | 4.35 | 0.05 | 0 | 0.00 | 0.00 | 1 | 1.02 | 0.01 |
| *Gamasellodes bicolor* | 5 | 4.35 | 0.22 | 2 | 4.55 | 0.06 | 9 | 17.39 | 0.42 | 0 | 0.00 | 0.00 | 16 | 6.12 | 0.10 |
| *Gamasellus montanus* | 0 | 0.00 | 0.00 | 1 | 4.55 | 0.03 | 0 | 0.00 | 0.00 | 1 | 3.33 | 0.01 | 2 | 2.04 | 0.01 |
| *Geholaspis longispinosus* | 0 | 0.00 | 0.00 | 0 | 0.00 | 0.00 | 0 | 0.00 | 0.00 | 1 | 3.33 | 0.01 | 1 | 1.02 | 0.01 |
| *Geholaspis mandibularis* | 0 | 0.00 | 0.00 | 0 | 0.00 | 0.00 | 0 | 0.00 | 0.00 | 1 | 3.33 | 0.01 | 1 | 1.02 | 0.01 |
| *Holoparasitus calcaratus* | 0 | 0.00 | 0.00 | 1 | 4.55 | 0.03 | 0 | 0.00 | 0.00 | 0 | 0.00 | 0.00 | 1 | 1.02 | 0.01 |
| *Hoploseius mariae* | 25 | 4.35 | 1.10 | 0 | 0.00 | 0.00 | 0 | 0.00 | 0.00 | 0 | 0.00 | 0.00 | 25 | 1.02 | 0.16 |
| *Hoploseius oblongus* | 121 | 30.43 | 5.32 | 8 | 18.18 | 0.25 | 0 | 0.00 | 0.00 | 1 | 3.33 | 0.01 | 130 | 12.24 | 0.81 |
| *Iphidonopsis dendrophilus* | 0 | 0.00 | 0.00 | 0 | 0.00 | 0.00 | 2 | 4.35 | 0.09 | 0 | 0.00 | 0.00 | 2 | 1.02 | 0.01 |
| *Lasioseius fimetorum* | 0 | 0.00 | 0.00 | 0 | 0.00 | 0.00 | 0 | 0.00 | 0.00 | 3 | 3.33 | 0.04 | 3 | 1.02 | 0.02 |
| *Lasioseius muricatus* | 49 | 26.09 | 2.15 | 55 | 13.64 | 1.69 | 0 | 0.00 | 0.00 | 0 | 0.00 | 0.00 | 104 | 9.18 | 0.65 |
| *Lasioseius ometes* | 24 | 30.43 | 1.05 | 24 | 27.27 | 0.74 | 6 | 8.70 | 0.28 | 61 | 13.33 | 0.73 | 115 | 19.39 | 0.72 |
| *Microgynium rectangulatum* | 0 | 0.00 | 0.00 | 0 | 0.00 | 0.00 | 3 | 4.35 | 0.14 | 5 | 16.67 | 0.06 | 8 | 6.12 | 0.05 |
| *Ololaelaps placentula* | 0 | 0.00 | 0.00 | 0 | 0.00 | 0.00 | 1 | 4.35 | 0.05 | 0 | 0.00 | 0.00 | 1 | 1.02 | 0.01 |
| *Parasitus* sp. | 0 | 0.00 | 0.00 | 0 | 0.00 | 0.00 | 0 | 0.00 | 0.00 | 1 | 3.33 | 0.01 | 1 | 1.02 | 0.01 |
| *Parazercon radiatus* | 0 | 0.00 | 0.00 | 0 | 0.00 | 0.00 | 1 | 4.35 | 0.05 | 0 | 0.00 | 0.00 | 1 | 1.02 | 0.01 |
| *Pergamasus* sp. | 0 | 0.00 | 0.00 | 0 | 0.00 | 0.00 | 0 | 0.00 | 0.00 | 1 | 3.33 | 0.01 | 1 | 1.02 | 0.01 |
| *Pleuronectocelaeno austriaca* | 0 | 0.00 | 0.00 | 2 | 4.55 | 0.06 | 1 | 4.35 | 0.05 | 2 | 6.67 | 0.02 | 5 | 4.08 | 0.03 |
| *Pneumolaelaps lubrica* | 2 | 8.70 | 0.09 | 0 | 0.00 | 0.00 | 5 | 8.70 | 0.23 | 7 | 16.67 | 0.08 | 14 | 9.18 | 0.09 |
| *Proctolaelaps fisheri* | 0 | 0.00 | 0.00 | 0 | 0.00 | 0.00 | 1 | 4.35 | 0.05 | 0 | 0.00 | 0.00 | 1 | 1.02 | 0.01 |
| *Proctolaelaps pygmaeus* | 0 | 0.00 | 0.00 | 0 | 0.00 | 0.00 | 0 | 0.00 | 0.00 | 1 | 3.33 | 0.01 | 1 | 1.02 | 0.01 |
| *Sejus polonicus* | 0 | 0.00 | 0.00 | 1 | 4.55 | 0.03 | 0 | 0.00 | 0.00 | 0 | 0.00 | 0.00 | 1 | 1.02 | 0.01 |
| *Sejus togatus* | 0 | 0.00 | 0.00 | 4 | 9.09 | 0.12 | 10 | 13.04 | 0.47 | 31 | 16.67 | 0.37 | 45 | 10.20 | 0.28 |
| *Trichouropoda ovalis* | 6 | 13.04 | 0.26 | 10 | 18.18 | 0.31 | 29 | 17.39 | 1.36 | 35 | 30.00 | 0.42 | 80 | 20.41 | 0.50 |
| *Uroobovella vinicolora* | 4 | 13.04 | 0.18 | 1 | 4.55 | 0.03 | 7 | 8.70 | 0.33 | 0 | 0.00 | 0.00 | 12 | 6.12 | 0.08 |
| *Veigaia kochi* | 0 | 0.00 | 0.00 | 0 | 0.00 | 0.00 | 0 | 0.00 | 0.00 | 2 | 6.67 | 0.02 | 2 | 2.04 | 0.01 |
| *Veigaia nemorensis* | 1 | 4.35 | 0.04 | 0 | 0.00 | 0.00 | 2 | 4.35 | 0.09 | 13 | 20.00 | 0.16 | 16 | 8.16 | 0.10 |
| *Veigaia transisale* | 0 | 0.00 | 0.00 | 0 | 0.00 | 0.00 | 2 | 4.35 | 0.09 | 5 | 10.00 | 0.06 | 7 | 4.08 | 0.04 |
| *Zercon curiosus* | 9 | 17.39 | 0.40 | 28 | 45.45 | 0.86 | 28 | 13.04 | 1.31 | 52 | 33.33 | 0.63 | 117 | 27.55 | 0.73 |
| *Zerconopsis decemremiger* | 3 | 8.70 | 0.13 | 12 | 4.55 | 0.37 | 0 | 0.00 | 0.00 | 1 | 3.33 | 0.01 | 16 | 4.08 | 0.10 |
| *Zerconopsis michaeli* | 0 | 0.00 | 0.00 | 1 | 4.55 | 0.03 | 0 | 0.00 | 0.00 | 0 | 0.00 | 0.00 | 1 | 1.02 | 0.01 |
| *Zerconopsis remiger* | 2 | 8.70 | 0.09 | 17 | 9.09 | 0.52 | 0 | 0.00 | 0.00 | 2 | 3.33 | 0.02 | 21 | 5.10 | 0.13 |
| **Acari, Oribatida** | 1677 | 86.96 | 73.68 | 2732 | 86.36 | 84.06 | 1480 | 91.30 | 69.48 | 6654 | 90.00 | 80.04 | 12543 | 88.78 | 78.55 |
| *Achipteria coleoptrata* | 4 | 8.70 | 0.18 | 4 | 13.64 | 0.12 | 3 | 8.70 | 0.14 | 11 | 13.33 | 0.13 | 22 | 11.22 | 0.14 |
| *Achipteria nitens* | 0 | 0.00 | 0.00 | 0 | 0.00 | 0.00 | 0 | 0.00 | 0.00 | 24 | 6.67 | 0.29 | 24 | 2.04 | 0.15 |
| *Achipteria* sp. | 6 | 17.39 | 0.26 | 1 | 4.55 | 0.03 | 3 | 8.70 | 0.14 | 28 | 13.33 | 0.34 | 38 | 11.22 | 0.24 |
| *Acrogalumna longipluma* | 0 | 0.00 | 0.00 | 0 | 0.00 | 0.00 | 2 | 4.35 | 0.09 | 3 | 10.00 | 0.04 | 5 | 4.08 | 0.03 |
| *Adoristes ovatus* | 0 | 0.00 | 0.00 | 1 | 4.55 | 0.03 | 0 | 0.00 | 0.00 | 0 | 0.00 | 0.00 | 1 | 1.02 | 0.01 |
| *Autogneta longilamellata* | 9 | 8.70 | 0.40 | 0 | 0.00 | 0.00 | 4 | 4.35 | 0.19 | 18 | 6.67 | 0.22 | 31 | 5.10 | 0.19 |
| *Autogneta* sp. | 0 | 0.00 | 0.00 | 0 | 0.00 | 0.00 | 0 | 0.00 | 0.00 | 1 | 3.33 | 0.01 | 1 | 1.02 | 0.01 |
| *Banksinoma lanceolata* | 0 | 0.00 | 0.00 | 0 | 0.00 | 0.00 | 0 | 0.00 | 0.00 | 1 | 3.33 | 0.01 | 1 | 1.02 | 0.01 |
| *Caleremaeus monilipes* | 2 | 8.70 | 0.09 | 0 | 0.00 | 0.00 | 0 | 0.00 | 0.00 | 2 | 6.67 | 0.02 | 4 | 4.08 | 0.03 |
| *Carabodes areolatus* | 22 | 8.70 | 0.97 | 4 | 18.18 | 0.12 | 4 | 4.35 | 0.19 | 11 | 20.00 | 0.13 | 41 | 13.27 | 0.26 |
| *Carabodes coriaceus* | 11 | 13.04 | 0.48 | 147 | 50.00 | 4.52 | 121 | 21.74 | 5.68 | 40 | 20.00 | 0.48 | 319 | 25.51 | 2.00 |
| *Carabodes femoralis* | 514 | 78.26 | 22.58 | 1481 | 77.27 | 45.57 | 881 | 60.87 | 41.36 | 5935 | 83.33 | 71.39 | 8811 | 75.51 | 55.18 |
| *Carabodes labyrinthicus* | 21 | 13.04 | 0.92 | 33 | 36.36 | 1.02 | 8 | 17.39 | 0.38 | 7 | 16.67 | 0.08 | 69 | 20.41 | 0.43 |
| *Carabodes ornatus* | 17 | 17.39 | 0.75 | 6 | 18.18 | 0.18 | 4 | 4.35 | 0.19 | 14 | 20.00 | 0.17 | 41 | 15.31 | 0.26 |
| *Carabodes reticulatus* | 5 | 13.04 | 0.22 | 2 | 4.55 | 0.06 | 0 | 0.00 | 0.00 | 4 | 6.67 | 0.05 | 11 | 6.12 | 0.07 |
| *Carabodes* sp. | 7 | 8.70 | 0.31 | 13 | 9.09 | 0.40 | 2 | 4.35 | 0.09 | 1 | 3.33 | 0.01 | 23 | 6.12 | 0.14 |
| *Carabodes subarcticus* | 774 | 69.57 | 34.01 | 687 | 63.64 | 21.14 | 227 | 73.91 | 10.66 | 201 | 66.67 | 2.42 | 1889 | 68.37 | 11.83 |
| *Carabodes tenuis* | 1 | 4.35 | 0.04 | 0 | 0.00 | 0.00 | 0 | 0.00 | 0.00 | 0 | 0.00 | 0.00 | 1 | 1.02 | 0.01 |
| *Cepheus cepheiformis* | 12 | 17.39 | 0.53 | 25 | 18.18 | 0.77 | 7 | 17.39 | 0.33 | 26 | 23.33 | 0.31 | 70 | 19.39 | 0.44 |
| *Cepheus dentatus* | 1 | 4.35 | 0.04 | 0 | 0.00 | 0.00 | 0 | 0.00 | 0.00 | 0 | 0.00 | 0.00 | 1 | 1.02 | 0.01 |
| *Cepheus* sp. | 1 | 4.35 | 0.04 | 0 | 0.00 | 0.00 | 0 | 0.00 | 0.00 | 1 | 3.33 | 0.01 | 2 | 2.04 | 0.01 |
| *Chamobates cuspidatus* | 24 | 30.43 | 1.05 | 40 | 27.27 | 1.23 | 6 | 13.04 | 0.28 | 17 | 26.67 | 0.20 | 87 | 24.49 | 0.54 |
| *Chamobates pusillus* | 4 | 8.70 | 0.18 | 0 | 0.00 | 0.00 | 0 | 0.00 | 0.00 | 0 | 0.00 | 0.00 | 4 | 2.04 | 0.03 |
| *Chamobates* sp. | 0 | 0.00 | 0.00 | 1 | 4.55 | 0.03 | 0 | 0.00 | 0.00 | 0 | 0.00 | 0.00 | 1 | 1.02 | 0.01 |
| *Chamobates spinosus* | 1 | 4.35 | 0.04 | 0 | 0.00 | 0.00 | 0 | 0.00 | 0.00 | 2 | 3.33 | 0.02 | 3 | 2.04 | 0.02 |
| *Chamobates voigtsi* | 0 | 0.00 | 0.00 | 0 | 0.00 | 0.00 | 0 | 0.00 | 0.00 | 2 | 6.67 | 0.02 | 2 | 2.04 | 0.01 |
| *Conchogneta dalecarlica* | 0 | 0.00 | 0.00 | 0 | 0.00 | 0.00 | 1 | 4.35 | 0.05 | 0 | 0.00 | 0.00 | 1 | 1.02 | 0.01 |
| *Conchogneta traegardhi* | 0 | 0.00 | 0.00 | 0 | 0.00 | 0.00 | 0 | 0.00 | 0.00 | 2 | 3.33 | 0.02 | 2 | 1.02 | 0.01 |
| *Cultroribula bicultrata* | 0 | 0.00 | 0.00 | 0 | 0.00 | 0.00 | 0 | 0.00 | 0.00 | 1 | 3.33 | 0.01 | 1 | 1.02 | 0.01 |
| *Cymbaeremaeus cymba* | 1 | 4.35 | 0.04 | 2 | 9.09 | 0.06 | 2 | 8.70 | 0.09 | 1 | 3.33 | 0.01 | 6 | 6.12 | 0.04 |
| *Damaeus* (*Adamaeus*) *onustus* | 3 | 4.35 | 0.13 | 0 | 0.00 | 0.00 | 2 | 4.35 | 0.09 | 4 | 10.00 | 0.05 | 9 | 5.10 | 0.06 |
| *Damaeus auritus* | 0 | 0.00 | 0.00 | 0 | 0.00 | 0.00 | 0 | 0.00 | 0.00 | 3 | 3.33 | 0.04 | 3 | 1.02 | 0.02 |
| *Damaeus crispatus* | 0 | 0.00 | 0.00 | 3 | 4.55 | 0.09 | 0 | 0.00 | 0.00 | 0 | 0.00 | 0.00 | 3 | 1.02 | 0.02 |
| *Damaeus* (*Paradamaeus*) *clavipes* | 0 | 0.00 | 0.00 | 0 | 0.00 | 0.00 | 2 | 4.35 | 0.09 | 1 | 3.33 | 0.01 | 3 | 2.04 | 0.02 |
| *Damaeus riparius* | 4 | 4.35 | 0.18 | 0 | 0.00 | 0.00 | 1 | 4.35 | 0.05 | 2 | 6.67 | 0.02 | 7 | 4.08 | 0.04 |
| *Damaeus* sp. | 13 | 13.04 | 0.57 | 17 | 27.27 | 0.52 | 6 | 17.39 | 0.28 | 13 | 23.33 | 0.16 | 49 | 20.41 | 0.31 |
| *Dissorhina ornata* | 70 | 21.74 | 3.08 | 4 | 9.09 | 0.12 | 2 | 8.70 | 0.09 | 9 | 16.67 | 0.11 | 85 | 14.29 | 0.53 |
| *Eniochthonius minutissimus* | 1 | 4.35 | 0.04 | 1 | 4.55 | 0.03 | 0 | 0.00 | 0.00 | 1 | 3.33 | 0.01 | 3 | 3.06 | 0.02 |
| *Epidamaeus bituberculatus* | 0 | 0.00 | 0.00 | 0 | 0.00 | 0.00 | 0 | 0.00 | 0.00 | 4 | 6.67 | 0.05 | 4 | 2.04 | 0.03 |
| *Epidamaeus setiger* | 1 | 4.35 | 0.04 | 0 | 0.00 | 0.00 | 0 | 0.00 | 0.00 | 1 | 3.33 | 0.01 | 2 | 2.04 | 0.01 |
| *Eremaeus tuberosus* | 0 | 0.00 | 0.00 | 0 | 0.00 | 0.00 | 4 | 4.35 | 0.19 | 0 | 0.00 | 0.00 | 4 | 1.02 | 0.03 |
| *Eueremaeus silvestris* | 12 | 4.35 | 0.53 | 1 | 4.55 | 0.03 | 10 | 17.39 | 0.47 | 1 | 3.33 | 0.01 | 24 | 7.14 | 0.15 |
| *Eupelops acromios* | 0 | 0.00 | 0.00 | 0 | 0.00 | 0.00 | 6 | 4.35 | 0.28 | 0 | 0.00 | 0.00 | 6 | 1.02 | 0.04 |
| *Eupelops hirtus* | 0 | 0.00 | 0.00 | 1 | 4.55 | 0.03 | 0 | 0.00 | 0.00 | 1 | 3.33 | 0.01 | 2 | 2.04 | 0.01 |
| *Euphthiracarus cribrarius* | 0 | 0.00 | 0.00 | 0 | 0.00 | 0.00 | 1 | 4.35 | 0.05 | 1 | 3.33 | 0.01 | 2 | 2.04 | 0.01 |
| *Euzetes globulus* | 0 | 0.00 | 0.00 | 0 | 0.00 | 0.00 | 0 | 0.00 | 0.00 | 1 | 3.33 | 0.01 | 1 | 1.02 | 0.01 |
| *Furcoribula furcillata* | 1 | 4.35 | 0.04 | 0 | 0.00 | 0.00 | 0 | 0.00 | 0.00 | 0 | 0.00 | 0.00 | 1 | 1.02 | 0.01 |
| *Fuscozetes setosus* | 2 | 8.70 | 0.09 | 0 | 0.00 | 0.00 | 2 | 4.35 | 0.09 | 0 | 0.00 | 0.00 | 4 | 3.06 | 0.03 |
| *Galumna* sp. | 3 | 4.35 | 0.13 | 0 | 0.00 | 0.00 | 1 | 4.35 | 0.05 | 1 | 3.33 | 0.01 | 5 | 3.06 | 0.03 |
| *Globozetes longipilus* | 0 | 0.00 | 0.00 | 0 | 0.00 | 0.00 | 1 | 4.35 | 0.05 | 5 | 10.00 | 0.06 | 6 | 4.08 | 0.04 |
| *Graptoppia foveolata* | 0 | 0.00 | 0.00 | 1 | 4.55 | 0.03 | 0 | 0.00 | 0.00 | 0 | 0.00 | 0.00 | 1 | 1.02 | 0.01 |
| *Hafenrefferia gilvipes* | 0 | 0.00 | 0.00 | 0 | 0.00 | 0.00 | 0 | 0.00 | 0.00 | 11 | 10.00 | 0.13 | 11 | 3.06 | 0.07 |
| *Hypochthonius rufulus* | 0 | 0.00 | 0.00 | 2 | 4.55 | 0.06 | 0 | 0.00 | 0.00 | 29 | 6.67 | 0.35 | 31 | 3.06 | 0.19 |
| *Kunstidamaeus tecticola* | 0 | 0.00 | 0.00 | 0 | 0.00 | 0.00 | 0 | 0.00 | 0.00 | 1 | 3.33 | 0.01 | 1 | 1.02 | 0.01 |
| *Lagenobates lagenulus* | 2 | 8.70 | 0.09 | 0 | 0.00 | 0.00 | 0 | 0.00 | 0.00 | 3 | 6.67 | 0.04 | 5 | 4.08 | 0.03 |
| *Liacarus coracinus* | 8 | 17.39 | 0.35 | 3 | 9.09 | 0.09 | 1 | 4.35 | 0.05 | 4 | 13.33 | 0.05 | 16 | 11.22 | 0.10 |
| *Licneremaeus licnophorus* | 0 | 0.00 | 0.00 | 0 | 0.00 | 0.00 | 0 | 0.00 | 0.00 | 1 | 3.33 | 0.01 | 1 | 1.02 | 0.01 |
| *Liebstadia longior* | 0 | 0.00 | 0.00 | 1 | 4.55 | 0.03 | 0 | 0.00 | 0.00 | 0 | 0.00 | 0.00 | 1 | 1.02 | 0.01 |
| *Malaconothrus monodactylus* | 1 | 4.35 | 0.04 | 0 | 0.00 | 0.00 | 0 | 0.00 | 0.00 | 2 | 3.33 | 0.02 | 3 | 2.04 | 0.02 |
| *Melanozetes mollicomus* | 15 | 8.70 | 0.66 | 0 | 0.00 | 0.00 | 0 | 0.00 | 0.00 | 2 | 6.67 | 0.02 | 17 | 4.08 | 0.11 |
| *Metabelba* sp. | 0 | 0.00 | 0.00 | 2 | 4.55 | 0.06 | 0 | 0.00 | 0.00 | 1 | 3.33 | 0.01 | 3 | 2.04 | 0.02 |
| *Multioppia gabra* | 0 | 0.00 | 0.00 | 0 | 0.00 | 0.00 | 0 | 0.00 | 0.00 | 3 | 3.33 | 0.04 | 3 | 1.02 | 0.02 |
| *Nanhermannia dorsalis* | 0 | 0.00 | 0.00 | 0 | 0.00 | 0.00 | 0 | 0.00 | 0.00 | 1 | 3.33 | 0.01 | 1 | 1.02 | 0.01 |
| *Nanhermannia nana* | 0 | 0.00 | 0.00 | 9 | 4.55 | 0.28 | 0 | 0.00 | 0.00 | 28 | 20.00 | 0.34 | 37 | 7.14 | 0.23 |
| *Neoliodes theleproctus* | 0 | 0.00 | 0.00 | 0 | 0.00 | 0.00 | 1 | 4.35 | 0.05 | 0 | 0.00 | 0.00 | 1 | 1.02 | 0.01 |
| *Neoribates aurantiacus* | 10 | 8.70 | 0.44 | 168 | 13.64 | 5.17 | 33 | 17.39 | 1.55 | 4 | 10.00 | 0.05 | 215 | 12.24 | 1.35 |
| *Nothrus silvestris* | 0 | 0.00 | 0.00 | 0 | 0.00 | 0.00 | 0 | 0.00 | 0.00 | 5 | 10.00 | 0.06 | 5 | 3.06 | 0.03 |
| *Oppia nitens* | 0 | 0.00 | 0.00 | 0 | 0.00 | 0.00 | 1 | 4.35 | 0.05 | 2 | 3.33 | 0.02 | 3 | 2.04 | 0.02 |
| *Oppiella* sp. | 4 | 13.04 | 0.18 | 2 | 4.55 | 0.06 | 0 | 0.00 | 0.00 | 1 | 3.33 | 0.01 | 7 | 5.10 | 0.04 |
| *Oppiella (Moritzoppia)keilbachi* | 0 | 0.00 | 0.00 | 0 | 0.00 | 0.00 | 0 | 0.00 | 0.00 | 5 | 10.00 | 0.06 | 5 | 3.06 | 0.03 |
| *Oppiella (Moritzoppia)unicarinata* | 1 | 4.35 | 0.04 | 6 | 4.55 | 0.18 | 0 | 0.00 | 0.00 | 10 | 6.67 | 0.12 | 17 | 4.08 | 0.11 |
| *Oppiella (Oppiella) falcata* | 0 | 0.00 | 0.00 | 0 | 0.00 | 0.00 | 0 | 0.00 | 0.00 | 1 | 3.33 | 0.01 | 1 | 1.02 | 0.01 |
| *Oppiella (Oppiella) nova* | 1 | 4.35 | 0.04 | 1 | 4.55 | 0.03 | 0 | 0.00 | 0.00 | 4 | 3.33 | 0.05 | 6 | 3.06 | 0.04 |
| *Oppiella (Oppiella)splendens* | 0 | 0.00 | 0.00 | 0 | 0.00 | 0.00 | 0 | 0.00 | 0.00 | 1 | 3.33 | 0.01 | 1 | 1.02 | 0.01 |
| *Oppiella (Rhinoppia)subpectinata* | 3 | 4.35 | 0.13 | 0 | 0.00 | 0.00 | 0 | 0.00 | 0.00 | 5 | 6.67 | 0.06 | 8 | 3.06 | 0.05 |
| *Oribatella calcarata* | 11 | 17.39 | 0.48 | 2 | 9.09 | 0.06 | 6 | 8.70 | 0.28 | 11 | 23.33 | 0.13 | 30 | 15.31 | 0.19 |
| *Oribatella sexdentata* | 5 | 8.70 | 0.22 | 0 | 0.00 | 0.00 | 1 | 4.35 | 0.05 | 8 | 16.67 | 0.10 | 14 | 8.16 | 0.09 |
| *Oribatella similesuperbula* | 0 | 0.00 | 0.00 | 0 | 0.00 | 0.00 | 0 | 0.00 | 0.00 | 2 | 3.33 | 0.02 | 2 | 1.02 | 0.01 |
| *Oribatula tibialis* | 0 | 0.00 | 0.00 | 5 | 9.09 | 0.15 | 0 | 0.00 | 0.00 | 0 | 0.00 | 0.00 | 5 | 2.04 | 0.03 |
| Other juveniles | 3 | 13.04 | 0.13 | 16 | 18.18 | 0.49 | 10 | 13.04 | 0.47 | 10 | 13.33 | 0.12 | 39 | 14.29 | 0.24 |
| *Parachipteria punctata* | 18 | 17.39 | 0.79 | 1 | 4.55 | 0.03 | 8 | 4.35 | 0.38 | 14 | 6.67 | 0.17 | 41 | 8.16 | 0.26 |
| *Parachipteria* sp. | 0 | 0.00 | 0.00 | 1 | 4.55 | 0.03 | 0 | 0.00 | 0.00 | 0 | 0.00 | 0.00 | 1 | 1.02 | 0.01 |
| *Pergalumna nervosa* | 0 | 0.00 | 0.00 | 4 | 4.55 | 0.12 | 5 | 4.35 | 0.23 | 3 | 6.67 | 0.04 | 12 | 4.08 | 0.08 |
| *Phauloppia nemoralis* | 7 | 13.04 | 0.31 | 0 | 0.00 | 0.00 | 1 | 4.35 | 0.05 | 3 | 10.00 | 0.04 | 11 | 7.14 | 0.07 |
| *Phthiracarus anonymus* | 0 | 0.00 | 0.00 | 1 | 4.55 | 0.03 | 0 | 0.00 | 0.00 | 1 | 3.33 | 0.01 | 2 | 2.04 | 0.01 |
| *Phthiracarus bryobius* | 0 | 0.00 | 0.00 | 0 | 0.00 | 0.00 | 0 | 0.00 | 0.00 | 2 | 6.67 | 0.02 | 2 | 2.04 | 0.01 |
| *Phthiracarus globosus* | 1 | 4.35 | 0.04 | 0 | 0.00 | 0.00 | 0 | 0.00 | 0.00 | 0 | 0.00 | 0.00 | 1 | 1.02 | 0.01 |
| *Phthiracarus compressus* | 1 | 4.35 | 0.04 | 3 | 9.09 | 0.09 | 0 | 0.00 | 0.00 | 5 | 6.67 | 0.06 | 9 | 5.10 | 0.06 |
| *Phthiracarus crinitus* | 0 | 0.00 | 0.00 | 0 | 0.00 | 0.00 | 0 | 0.00 | 0.00 | 2 | 3.33 | 0.02 | 2 | 1.02 | 0.01 |
| *Phthiracarus ferrugineus* | 0 | 0.00 | 0.00 | 0 | 0.00 | 0.00 | 0 | 0.00 | 0.00 | 2 | 3.33 | 0.02 | 2 | 1.02 | 0.01 |
| *Phthiracarus globosus* | 1 | 4.35 | 0.04 | 0 | 0.00 | 0.00 | 0 | 0.00 | 0.00 | 0 | 0.00 | 0.00 | 1 | 1.02 | 0.01 |
| *Phthiracarus longulus* | 2 | 8.70 | 0.09 | 1 | 4.55 | 0.03 | 2 | 8.70 | 0.09 | 11 | 16.67 | 0.13 | 16 | 10.20 | 0.10 |
| *Pilogalumna tenuiclava* | 1 | 4.35 | 0.04 | 1 | 4.55 | 0.03 | 0 | 0.00 | 0.00 | 0 | 0.00 | 0.00 | 2 | 2.04 | 0.01 |
| *Platynothrus peltifer* | 6 | 8.70 | 0.26 | 0 | 0.00 | 0.00 | 0 | 0.00 | 0.00 | 2 | 6.67 | 0.02 | 8 | 4.08 | 0.05 |
| *Platyliodes Scaliger* | 0 | 0.00 | 0.00 | 0 | 0.00 | 0.00 | 0 | 0.00 | 0.00 | 1 | 3.33 | 0.01 | 1 | 1.02 | 0.01 |
| *Porobelba spinosa* | 0 | 0.00 | 0.00 | 0 | 0.00 | 0.00 | 1 | 4.35 | 0.05 | 1 | 3.33 | 0.01 | 2 | 2.04 | 0.01 |
| *Quadroppia quadricarinata* | 1 | 4.35 | 0.04 | 0 | 0.00 | 0.00 | 0 | 0.00 | 0.00 | 0 | 0.00 | 0.00 | 1 | 1.02 | 0.01 |
| *Ramusella clavipectinata* | 0 | 0.00 | 0.00 | 0 | 0.00 | 0.00 | 2 | 8.70 | 0.09 | 4 | 6.67 | 0.05 | 6 | 4.08 | 0.04 |
| *Ramusella furcata* | 0 | 0.00 | 0.00 | 0 | 0.00 | 0.00 | 0 | 0.00 | 0.00 | 1 | 3.33 | 0.01 | 1 | 1.02 | 0.01 |
| *Scheloribates pallidulus* | 3 | 4.35 | 0.13 | 2 | 9.09 | 0.06 | 29 | 21.74 | 1.36 | 10 | 20.00 | 0.12 | 44 | 14.29 | 0.28 |
| *Scheloribates latipes* | 0 | 0.00 | 0.00 | 0 | 0.00 | 0.00 | 22 | 17.39 | 1.03 | 3 | 10.00 | 0.04 | 25 | 7.14 | 0.16 |
| *Scheloribates* sp. | 0 | 0.00 | 0.00 | 0 | 0.00 | 0.00 | 1 | 4.35 | 0.05 | 0 | 0.00 | 0.00 | 1 | 1.02 | 0.01 |
| *Spatiodamaeus boreus* | 0 | 0.00 | 0.00 | 0 | 0.00 | 0.00 | 3 | 4.35 | 0.14 | 1 | 3.33 | 0.01 | 4 | 2.04 | 0.03 |
| *Spatiodamaeus fageti* | 0 | 0.00 | 0.00 | 0 | 0.00 | 0.00 | 3 | 4.35 | 0.14 | 0 | 0.00 | 0.00 | 3 | 1.02 | 0.02 |
| *Spatiodamaeus* sp. | 0 | 0.00 | 0.00 | 0 | 0.00 | 0.00 | 1 | 4.35 | 0.05 | 0 | 0.00 | 0.00 | 1 | 1.02 | 0.01 |
| *Steganacarus (Atropacarus)striculus* | 0 | 0.00 | 0.00 | 1 | 4.55 | 0.03 | 0 | 0.00 | 0.00 | 0 | 0.00 | 0.00 | 1 | 1.02 | 0.01 |
| *Steganacarus(Steganacarus) magnus* | 1 | 4.35 | 0.04 | 0 | 0.00 | 0.00 | 1 | 4.35 | 0.05 | 0 | 0.00 | 0.00 | 2 | 2.04 | 0.01 |
| *Steganacarus* (*Tropacarus*) *carinatus* | 0 | 0.00 | 0.00 | 0 | 0.00 | 0.00 | 0 | 0.00 | 0.00 | 2 | 3.33 | 0.02 | 2 | 1.02 | 0.01 |
| *Subiasella quadrimaculata* | 0 | 0.00 | 0.00 | 14 | 9.09 | 0.43 | 0 | 0.00 | 0.00 | 6 | 13.33 | 0.07 | 20 | 6.12 | 0.13 |
| *Suctobelba atomaria* | 5 | 4.35 | 0.22 | 0 | 0.00 | 0.00 | 0 | 0.00 | 0.00 | 2 | 3.33 | 0.02 | 7 | 2.04 | 0.04 |
| *Tectocepheus velatus alatus* | 11 | 4.35 | 0.48 | 7 | 9.09 | 0.22 | 3 | 13.04 | 0.14 | 2 | 3.33 | 0.02 | 23 | 7.14 | 0.14 |
| *Tectocepheus velatus velatus* | 0 | 0.00 | 0.00 | 1 | 4.55 | 0.03 | 2 | 4.35 | 0.09 | 0 | 0.00 | 0.00 | 3 | 2.04 | 0.02 |
| *Tectocepheus* sp. | 1 | 4.35 | 0.04 | 0 | 0.00 | 0.00 | 1 | 4.35 | 0.05 | 0 | 0.00 | 0.00 | 2 | 2.04 | 0.01 |
| *Xenillus tegeocranus* | 2 | 4.35 | 0.09 | 0 | 0.00 | 0.00 | 0 | 0.00 | 0.00 | 2 | 6.67 | 0.02 | 4 | 3.06 | 0.03 |
| *Zygoribatula exilis* | 5 | 8.70 | 0.22 | 3 | 13.64 | 0.09 | 26 | 8.70 | 1.22 | 17 | 13.33 | 0.20 | 51 | 11.22 | 0.32 |
| **Collembola** | 57 | 47.83 | 2.50 | 24 | 50.00 | 0.74 | 26 | 43.48 | 1.22 | 212 | 66.67 | 2.55 | 319 | 53.06 | 2.00 |
| *Arrhopalites coecus* | 0 | 0.00 | 0.00 | 0 | 0.00 | 0.00 | 0 | 0.00 | 0.00 | 2 | 3.33 | 0.02 | 2 | 1.02 | 0.01 |
| *Caprainea marginata* | 6 | 13.04 | 0.26 | 6 | 18.18 | 0.18 | 5 | 4.35 | 0.23 | 25 | 20.00 | 0.30 | 42 | 14.29 | 0.26 |
| *Cyphoderus albinus* | 0 | 0.00 | 0.00 | 0 | 0.00 | 0.00 | 0 | 0.00 | 0.00 | 6 | 6.67 | 0.07 | 6 | 2.04 | 0.04 |
| *Entomobrya arborea* | 2 | 4.35 | 0.09 | 0 | 0.00 | 0.00 | 1 | 4.35 | 0.05 | 5 | 6.67 | 0.06 | 8 | 4.08 | 0.05 |
| *Entomobrya corticalis* | 19 | 30.43 | 0.83 | 4 | 18.18 | 0.12 | 14 | 21.74 | 0.66 | 97 | 46.67 | 1.17 | 134 | 30.61 | 0.84 |
| *Entomobrya marginata* | 0 | 0.00 | 0.00 | 0 | 0.00 | 0.00 | 0 | 0.00 | 0.00 | 1 | 3.33 | 0.01 | 1 | 1.02 | 0.01 |
| *Entomobrya multifasciata* | 0 | 0.00 | 0.00 | 0 | 0.00 | 0.00 | 1 | 4.35 | 0.05 | 0 | 0.00 | 0.00 | 1 | 1.02 | 0.01 |
| *Entomobrya muscorum* | 0 | 0.00 | 0.00 | 1 | 4.55 | 0.03 | 0 | 0.00 | 0.00 | 6 | 6.67 | 0.07 | 7 | 3.06 | 0.04 |
| *Entomobrya nivalis* | 0 | 0.00 | 0.00 | 0 | 0.00 | 0.00 | 0 | 0.00 | 0.00 | 7 | 10.00 | 0.08 | 7 | 3.06 | 0.04 |
| *Lathriopyga stachi* | 0 | 0.00 | 0.00 | 0 | 0.00 | 0.00 | 0 | 0.00 | 0.00 | 1 | 3.33 | 0.01 | 1 | 1.02 | 0.01 |
| *Lepidocyrtus cyaneus* | 5 | 8.70 | 0.22 | 6 | 9.09 | 0.18 | 0 | 0.00 | 0.00 | 16 | 13.33 | 0.19 | 27 | 8.16 | 0.17 |
| *Orchesella bifasciata* | 0 | 0.00 | 0.00 | 2 | 9.09 | 0.06 | 0 | 0.00 | 0.00 | 0 | 0.00 | 0.00 | 2 | 2.04 | 0.01 |
| *Pseudachorutella assigilata* | 0 | 0.00 | 0.00 | 2 | 4.55 | 0.06 | 1 | 4.35 | 0.05 | 0 | 0.00 | 0.00 | 3 | 2.04 | 0.02 |
| *Pseudisotoma sensibilis* | 14 | 13.04 | 0.62 | 0 | 0.00 | 0.00 | 1 | 4.35 | 0.05 | 4 | 6.67 | 0.05 | 19 | 6.12 | 0.12 |
| *Pseudosinella immaculata* | 1 | 4.35 | 0.04 | 2 | 4.55 | 0.06 | 0 | 0.00 | 0.00 | 0 | 0.00 | 0.00 | 3 | 2.04 | 0.02 |
| *Sinella coeca* | 1 | 4.35 | 0.04 | 0 | 0.00 | 0.00 | 0 | 0.00 | 0.00 | 6 | 6.67 | 0.07 | 7 | 3.06 | 0.04 |
| *Sinella myrmecophila* | 2 | 4.35 | 0.09 | 1 | 4.55 | 0.03 | 0 | 0.00 | 0.00 | 0 | 0.00 | 0.00 | 3 | 2.04 | 0.02 |
| *Tomocerus minor* | 7 | 13.04 | 0.31 | 0 | 0.00 | 0.00 | 3 | 8.70 | 0.14 | 35 | 30.00 | 0.42 | 45 | 14.29 | 0.28 |
| *Vertagopus cinerea* | 0 | 0.00 | 0.00 | 0 | 0.00 | 0.00 | 0 | 0.00 | 0.00 | 1 | 3.33 | 0.01 | 1 | 1.02 | 0.01 |
| **Insecta** | 320 | 78.26 | 13.47 | 251 | 77.27 | 7.72 | 407 | 91.30 | 19.12 | 717 | 90.00 | 8.63 | 1695 | 84.69 | 10.55 |
| *Acrotrichis* sp. | 0 | 0.00 | 0.00 | 0 | 0.00 | 0.00 | 0 | 0.00 | 0.00 | 1 | 3.33 | 0.01 | 1 | 1.02 | 0.01 |
| *Attagenus* sp. | 0 | 0.00 | 0.00 | 0 | 0.00 | 0.00 | 1 | 4.35 | 0.05 | 0 | 0.00 | 0.00 | 1 | 1.02 | 0.01 |
| *Bolitophagus reticulatus* | 0 | 0.00 | 0.00 | 0 | 0.00 | 0.00 | 1 | 4.35 | 0.05 | 0 | 0.00 | 0.00 | 1 | 1.02 | 0.01 |
| *Cerylon fagi* | 0 | 0.00 | 0.00 | 0 | 0.00 | 0.00 | 0 | 0.00 | 0.00 | 1 | 3.33 | 0.01 | 1 | 1.02 | 0.01 |
| *Cerylon ferrugineum* | 0 | 0.00 | 0.00 | 0 | 0.00 | 0.00 | 0 | 0.00 | 0.00 | 1 | 3.33 | 0.01 | 1 | 1.02 | 0.01 |
| *Cerylon* sp. | 0 | 0.00 | 0.00 | 0 | 0.00 | 0.00 | 0 | 0.00 | 0.00 | 2 | 6.67 | 0.02 | 2 | 2.04 | 0.01 |
| Ciidae | 0 | 0.00 | 0.00 | 1 | 4.55 | 0.03 | 4 | 8.70 | 0.19 | 1 | 3.33 | 0.01 | 6 | 4.08 | 0.04 |
| *Cis* spp. | 47 | 30.43 | 2.07 | 230 | 50.00 | 7.08 | 243 | 78.26 | 11.41 | 646 | 83.33 | 7.77 | 1166 | 62.24 | 7.30 |
| Coleoptera | 69 | 21.74 | 3.03 | 0 | 0.00 | 0.00 | 5 | 4.35 | 0.23 | 10 | 10.00 | 0.12 | 84 | 9.18 | 0.53 |
| *Corticaria* sp. | 0 | 0.00 | 0.00 | 0 | 0.00 | 0.00 | 1 | 4.35 | 0.05 | 0 | 0.00 | 0.00 | 1 | 1.02 | 0.01 |
| *Cyphon* sp. | 1 | 4.35 | 0.04 | 0 | 0.00 | 0.00 | 0 | 0.00 | 0.00 | 0 | 0.00 | 0.00 | 1 | 1.02 | 0.01 |
| Dermaptera | 0 | 0.00 | 0.00 | 1 | 4.55 | 0.03 | 0 | 0.00 | 0.00 | 0 | 0.00 | 0.00 | 1 | 1.02 | 0.01 |
| Dermestidae | 0 | 0.00 | 0.00 | 0 | 0.00 | 0.00 | 1 | 4.35 | 0.05 | 0 | 0.00 | 0.00 | 1 | 1.02 | 0.01 |
| Diptera | 137 | 28.00 | 5.77 | 2 | 9.09 | 0.06 | 9 | 17.39 | 0.42 | 27 | 33.33 | 0.32 | 175 | 23.47 | 1.09 |
| Elateridae | 0 | 0.00 | 0.00 | 5 | 9.09 | 0.15 | 1 | 4.35 | 0.05 | 19 | 33.33 | 0.23 | 25 | 13.27 | 0.16 |
| *Ennearthron* spp. | 0 | 0.00 | 0.00 | 1 | 4.55 | 0.03 | 8 | 13.04 | 0.38 | 0 | 0.00 | 0.00 | 9 | 4.08 | 0.06 |
| Heteroptera | 0 | 0.00 | 0.00 | 0 | 0.00 | 0.00 | 1 | 4.35 | 0.05 | 0 | 0.00 | 0.00 | 1 | 1.02 | 0.01 |
| Hymenoptera | 3 | 13.04 | 0.13 | 3 | 13.64 | 0.09 | 2 | 8.70 | 0.09 | 1 | 3.33 | 0.01 | 9 | 9.18 | 0.06 |
| *Nargus velox* | 0 | 0.00 | 0.00 | 1 | 4.55 | 0.03 | 0 | 0.00 | 0.00 | 0 | 0.00 | 0.00 | 1 | 1.02 | 0.01 |
| *Octotemnus* spp. | 61 | 17.39 | 2.68 | 0 | 0.00 | 0.00 | 125 | 8.70 | 5.87 | 0 | 0.00 | 0.00 | 186 | 6.12 | 1.16 |
| Psocoptera | 0 | 0.00 | 0.00 | 2 | 4.55 | 0.06 | 0 | 0.00 | 0.00 | 0 | 0.00 | 0.00 | 2 | 1.02 | 0.01 |
| Ptinidae | 0 | 0.00 | 0.00 | 0 | 0.00 | 0.00 | 1 | 4.35 | 0.05 | 2 | 3.33 | 0.02 | 3 | 2.04 | 0.02 |
| *Ropalodontus* sp. | 0 | 0.00 | 0.00 | 0 | 0.00 | 0.00 | 2 | 4.35 | 0.09 | 0 | 0.00 | 0.00 | 2 | 1.02 | 0.01 |
| Staphylinidae | 1 | 4.35 | 0.04 | 3 | 13.64 | 0.09 | 2 | 4.35 | 0.09 | 5 | 13.33 | 0.06 | 11 | 9.18 | 0.07 |
| Thysanoptera | 1 | 4.35 | 0.04 | 2 | 4.55 | 0.06 | 0 | 0.00 | 0.00 | 0 | 0.00 | 0.00 | 3 | 2.04 | 0.02 |
| *Trixagus* sp. | 0 | 0.00 | 0.00 | 0 | 0.00 | 0.00 | 0 | 0.00 | 0.00 | 1 | 3.33 | 0.01 | 1 | 1.02 | 0.01 |
| **Total** | 2376 | 23.47 | 14.81 | 3250 | 22.45 | 20.35 | 2129 | 23.47 | 13.34 | 8313 | 30.61 | 52.06 | 16068 | 100.00 | 100.00 |

Appendix. Invertebrates occuring on fruiting bodies of bracket fungi in Białowieża National Park, Poland, from the four types of DD, in period June to August in 2014. Abu – abundance, Fre – frequency, Dom – Dominance.
